# Association between cigarette smoking and the risk of major psychiatric disorders: a systematic review and meta-analysis in depression, schizophrenia, and bipolar disorder

**DOI:** 10.3389/fmed.2025.1529191

**Published:** 2025-02-13

**Authors:** Zhonghou Hu, Enxiu Cui, Bo Chen, Mi Zhang

**Affiliations:** Third Department of Psychiatry, Yancheng Fourth People's Hospital, Yancheng, China

**Keywords:** psychiatric disorders, depression, schizophrenia, bipolar disorder, meta-analysis, cigarette smoking

## Abstract

**Background:**

The prevalence of cigarette smoking among patients with major psychiatric conditions is significantly higher than that in the general population. However, whether there is a causal association between cigarette smoking and major psychiatric disorders remains unclear. Therefore, we conducted a systematic review and meta-analysis of cohort studies to elucidate the association between cigarette smoking and the risk of major psychiatric disorders, including depression, schizophrenia, and bipolar disorder.

**Methods:**

We systematically searched PubMed, Embase, and the Cochrane Library for potentially eligible studies from their inception until March 2, 2024. All pooled analyses were performed using a random-effects model, and exploratory sensitivity and subgroup analyses were conducted.

**Results:**

Twenty-five cohort studies involving 2,917,030 individuals were included in the meta-analysis. The summary results indicated that both current smoking (relative risk [RR], 1.30; 95% confidence interval [CI], 1.18–1.43; *P* < 0.001) and former smoking (RR, 1.16; 95% CI, 1.09–1.23; *P* < 0.001) were associated with an elevated risk of major depression. Additionally, current smoking was significantly associated with an elevated risk of schizophrenia (RR, 1.84; 95% CI, 1.07–3.19; *P* = 0.028) and bipolar disorder (RR, 1.54; 95% CI, 1.22–1.95; *P* < 0.001).

**Conclusion:**

Current smoking is significantly associated with an elevated risk of major psychiatric disorders, including major depression, schizophrenia, and bipolar disorder. Former smokers also have an elevated risk of major depression. However, it should be noted that, despite these significant associations, due to the nature of the cohort studies included, this study cannot establish a causal relationship between cigarette smoking and major psychiatric disorders.

**Systematic review registration:**

https://inplasy.com/inplasy-2024-3-0093/, identifier INPLASY202430093.

## Introduction

Psychiatric disorders are a major cause of global disability, affecting an estimated 12.6% of the world's population and representing 14.6% of disability-adjusted life years (1). Currently, nearly two-thirds of individuals with psychiatric disorders experience their first symptoms before the age of 25, with a median age of onset of 18 years (2). In this study, we specifically focus on three major psychiatric disorders: depression, schizophrenia, and bipolar disorder. These disorders are classified as severe by authoritative systems like the Diagnostic and Statistical Manual of Mental Disorders and the International Classification of Diseases (3). They are characterized by a long-term course that meets diagnostic criteria and causes significant impairment in daily functioning. Specifically, depression, schizophrenia, and bipolar disorder are closely associated with adverse health outcomes, widespread disruptions in physical and mental functioning, and pose substantial challenges to public health (4).

Adults with psychiatric health issues face a greater risk of smoking-related diseases and mortality compared to those without psychiatric health problems (5). The higher cigarette smoking rates among adults with psychiatric health problems can be attributed to several factors, including targeted marketing by the tobacco industry (6). In adults with psychiatric health issues, the cigarette smoking rates are as follows: 40.8% for severe psychological distress (7), 33.3% for any psychiatric illness in the past year (8), 30.5% for major depressive disorder (9), and 26.9% for frequently feeling depressed (10). The exact mechanisms underlying the association between smoking and these major psychiatric disorders are complex and not yet fully understood. However, several lines of research have provided valuable insights into the possible physiological pathways. Nicotine, the primary active ingredient in tobacco, plays a crucial role. It stimulates the release of dopamine in the brain. Prolonged smoking can lead to dysregulation of the dopamine system, which is integral to emotional regulation. Disruptions in this system may exacerbate or contribute to the development of psychiatric disorders. For instance, an imbalance in dopamine signaling has been implicated in the pathophysiology of schizophrenia, where abnormal dopamine release in certain brain regions can lead to symptoms such as hallucinations and delusions (11). Smoking also releases inflammatory mediators such as TNF-α, IL-6, and C-reactive protein. These mediators can penetrate the blood-brain barrier, activating microglia, the brain's immune defense cells. This activation leads to neuroinflammation, which disrupts neuronal function, damages synaptic connections, and interferes with neural signal transmission. Moreover, smoking generates a high abundance of free radicals, initiating oxidative stress. The brain's natural defense mechanisms struggle to counteract the excess free radicals, resulting in neuronal damage and death. This impairment affects brain regions responsible for emotion, memory, and decision - making, and long - term oxidative stress is a significant contributor to the development of psychiatric disorders (12). In addition, unhealthy lifestyle factors often associated with smoking can lead to elevated levels of inflammation and increased oxidative stress in the body. Moreover, prolonged exposure to high - stress environments or experiencing childhood trauma can impact the structure and function of the brain. These impacts include reduced hippocampal volume and diminished prefrontal cortex function, which are involved in emotional regulation, memory, and decision - making processes. Consequently, these changes heighten the risk of developing psychiatric disorders (13). The complex interplay between smoking and these factors may contribute to the development and progression of major psychiatric disorders.

Several systematic reviews and meta-analyses have addressed the association between cigarette smoking status and the risk of major psychiatric disorders (14–16). Luger et al. identified 85 studies and found that smoking was associated with a nearly twofold increase in the risk of depression compared with never and former smokers. In smaller prospective studies, current smokers had a higher likelihood of developing depression than never smokers (14). Hunter et al. systematically searched Medline, Embase, PsychInfo, Maternity and Infant Care, and Web of Science and identified 12 studies (nine cohort and three case-control studies), indicating that smoking and prenatal smoke exposure may be independent risk factors for schizophrenia (15). Cerimele et al. identified six studies and found that tobacco use was significantly associated with the severity and symptoms of bipolar disorder and reduced functional levels (16). However, these studies have several limitations: (1) the analyses included cross-sectional studies, which cannot prove a causal association between smoking status and the risk of major psychiatric conditions; (2) the included populations did not exclude individuals with pre-existing major psychiatric disorders at the outset; (3) the causal association between smoking and major psychiatric disorders is not strictly stated, and there are cases where patients with major psychiatric disorders initiated smoking behavior; and (4) previous studies lacked in-depth exploratory analysis and did not examine the strength of the association between smoking and major psychiatric disorders in different populations. In the present study, we chose to conduct a systematic review and meta-analysis of cohort studies to elucidate the association between cigarette smoking status and the risk of major psychiatric disorders, including major depression, schizophrenia, and bipolar disorder. Cohort studies are particularly advantageous in this context as they allow us to follow participants over time, enabling us to observe the temporal sequence of events. This temporal dimension is crucial for understanding the potential exposure-outcome relationship, which is essential in epidemiological research. However, it is important to acknowledge that cohort studies also present challenges in establishing causation. Although they can provide evidence of association, they are subject to certain limitations. These include the potential for residual confounding, selection bias, and the influence of unmeasured confounding factors. Despite these challenges, we believe that cohort studies offer valuable insights into the natural history of the relationship between smoking and psychiatric disorders.

## Methods

### Data sources, search strategy, and selection criteria

This review was reported according to the guidelines outlined in the Preferred Reporting Items for Systematic Reviews and Meta-Analysis (PRISMA) Statement (17). The study was registered on the INPLASY platform (registration number: INPLASY202430093). Any cohort study investigating the association between cigarette smoking and major psychiatric disorders that met the inclusion criteria, with no restrictions on language or publication status (published, in press, or ongoing), was eligible. We conducted a comprehensive search of the PubMed, Embase, and Cochrane Library electronic databases for articles published through March 2, 2024, and used (“bipolar disorder” OR “bipolar” OR “affective disorder”) OR (“major depression” OR “depressive”) OR (“psychosis” OR “schizophrenia” OR “schizophrenic” OR “psychotic”) AND (“smoking” OR “cigarettes” OR “tobacco”) as the search terms. The full search strategy is provided in Supplementary material 1. To identify additional eligible studies, we manually searched the reference lists of all relevant original and review articles. We also used medical subject headings, methods, patient populations, study designs, exposures, and outcome variables from these articles to identify relevant studies.

Two independent reviewers screened the titles and abstracts of the identified studies to determine their eligibility. Full-text articles of potentially relevant studies were then retrieved and assessed for final inclusion. Any discrepancies between the reviewers were resolved through discussion or, if necessary, by consulting a third reviewer. Studies meeting the following criteria were included: (1) participants: general population initially without major psychiatric conditions; (2) exposure: current or former smoking. In each of the included studies, the definition of “current smoking” typically referred to individuals who were actively smoking cigarettes at the time of the study initiation. However, specific definitions varied among studies. “Former smoking” generally denoted individuals who had smoked cigarettes in the past but had ceased smoking at the start of the study; (3) control: nonsmoking; (4) outcome: effect estimates (odds ratio [OR], relative risk [RR], or hazard ratio [HR] and 95% confidence interval [CI]) for the association between cigarette smoking and major psychiatric disorders, including major depression, schizophrenia, and bipolar disorder; and (5) study design: cohort design. In studies reporting several multivariate-adjusted effect estimates, we extracted one effect estimate derived from the statistical model including the most covariates (18). Cross-sectional and case-control studies were excluded because a causal relationship between smoking and the development of major psychiatric disorders could not be demonstrated.

### Data collection and quality assessment

The collected data for each included study included the following information: first author's name, publication year, country, study design, setting, sample size, age at baseline, percentage of male patients, exposure categories, reported outcomes and assessment, effect estimate (OR, RR, or HR), follow-up duration, adjusted factors, reported effect estimate with 95%CI. Quality assessment of the included studies was conducted using the Newcastle-Ottawa Scale (NOS). The NOS is a tool that has been partially validated for the quality assessment of observational studies in meta-analyses. It evaluates studies based on three main components: selection (four items), comparability (one item), and outcome (three items). Each component is scored, with a total possible score ranging from 0 to 9 points. Higher scores indicate better methodological quality (19). Two independent reviewers extracted the data from the included studies using a standardized data extraction form, and the same two reviewers independently assessed the methodological quality of each study using the NOS. In case of any discrepancies between the two reviewers, a third reviewer was consulted. The third reviewer referred to the original study for an independent review and adjudication to resolve the disagreement.

### Statistical analyses

To investigate the association between cigarette smoking and the risk of major psychiatric disorders, we analyzed the effect estimates (OR, RR, or HR) and their 95%CI reported in each included study. The combined RR with 95% CI was calculated using a random-effects model, which accounts for the variability among the included studies (20). Statistical heterogeneity was evaluated using the *I*^2^ statistic and Cochran's Q test. Significant heterogeneity was defined as an *I*^2^ value >50.0% or a *P*-value < 0.10 (21, 22). The robustness of the pooled RR with 95% CI was assessed using sensitivity analysis by sequentially excluding a single study from the overall analysis (23). This approach helps to identify any individual studies that may have a disproportionate influence on the overall results. Subgroup analyses were performed to explore potential sources of heterogeneity and to provide more detailed insights into the association between smoking and major psychiatric disorders. Subgroup analyses were performed for major psychiatric disorders, including major depression, schizophrenia, and bipolar disorder, according to study design, sex, smoking intensity, follow-up duration, adjusted levels, and study quality, and the differences between subgroups were compared using the interaction *t*-test, which assumes that the data are normally distributed (24). A study adjusted for ≥3 factors was considered a high-adjusted level, whereas a study adjusted for one or two factors was considered a low-adjusted level. Publication bias was evaluated through a combination of qualitative and quantitative approaches, which included analysis of funnel plots and Egger and Begg tests (25, 26). All *P* values reported in this study were two-sided, with a significance level of 0.05. Statistical analyses were performed using the Stata software (version 12.0; StataCorp, College Station, Texas, USA).

## Results

### Search results

**Figure 1:**
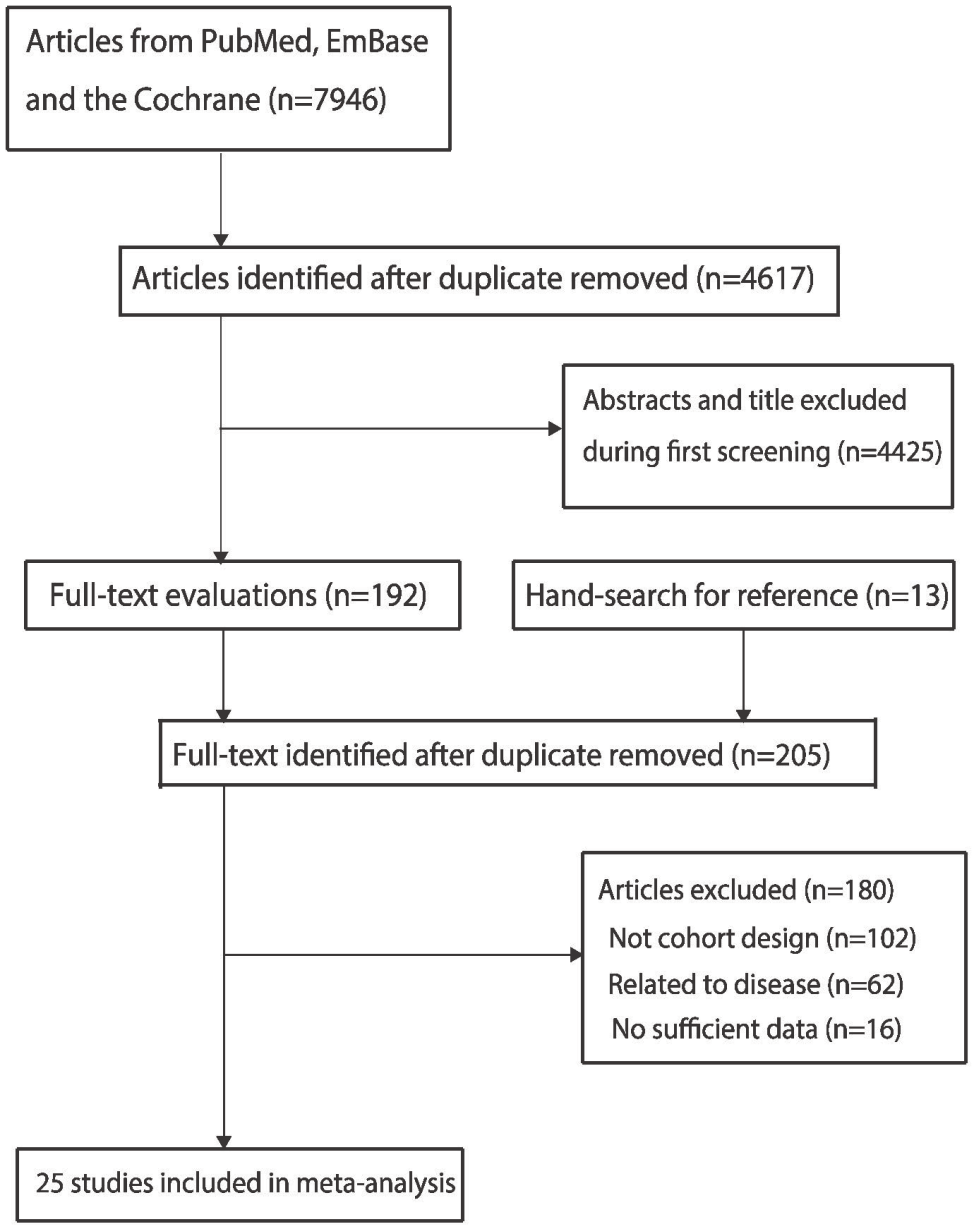
Details regarding literature search and study selection.

A total of 7,946 literature records were identified through the electronic search. After removing duplicate articles, 4,617 studies remained. Based on titles and abstracts, 4,425 studies were excluded due to irrelevant content, this left 192 full-text studies for further review. After a detailed full-text review, 167 studies were excluded for the following reasons: lack of cohort design (*n* = 89), related to other diseases (*n* = 62), and insufficient data (*n* = 16). The reference lists of the remaining studies were reviewed, yielding 13 potentially relevant studies, all 13 of these studies were excluded because they lacked a cohort design (Supplementary material 2). Finally, 25 cohort studies were selected for inclusion in the meta-analysis (Figure 1) (27–51).

### Study characteristics

The characteristics of the included studies and their participants are summarized in Table 1. In total, 2,917,030 individuals were included, and the sample size ranged from 671 to 1,647,728. Twenty-one studies were designed as prospective cohort studies, and the remaining four studies were designed as retrospective cohort studies. Nineteen studies reported the association between smoking status and major depression risk, six studies reported the association between smoking status and schizophrenia risk, and three studies reported bipolar disorder related to smoking status. The follow-up duration of included studies ranged from 1.5 years to 40.0 years. Individual study quality was evaluated based on the NOS, with five studies scoring 8, 12 scoring 7, and eight scoring 6 (Supplementary material 3).

**Table 1:** The baseline characteristics of identified studies and involved individuals.

**Study**	**Country**	**Study design**	**Setting**	**Sample size**	**Age (years)**	**Male (%)**	**Exposure categories**	**Reported outcomes**	**Effect estimate**	**Follow-up duration (years)**	**Adjusted factors**	**Study quality**
Breslau et al. (27)	USA	Prospective	Members of a large health maintenance organization in southeastern Michigan	974	21–30	38.0	Current smoking (smoking daily for 1 or more months)	Major depression (DSM-III-R)	OR	5.0	Early conduct problems and alcohol use disorder	8
Murphy et al. (28)	USA	Prospective	Gathered in a country in Atlantic Canada	2,204	NA	47.8	Current smoking (habits and amount)	Major depression (DPAX)	RR	18.0-22.0	Year of study, are controlled for age and gender	6
Zammit et al. (29)	UK	Prospective	Swedish army	50,053	18–20	100.0	Current smoking (amount)	Schizophrenia (ICD-8)	HR	27.0	Diagnosis at conscription, poor social integration, IQ, drug use, disturbed behavior, father's occupation, place of upbringing, family socioeconomic status, family psychiatric history, and alcohol problems	7
Weiser et al. (30)	USA	Prospective	Israel Defense Forces Medical Corps	14,248	NA	100.0	Current smoking (habits and amount)	Schizophrenia (ICD-9, ICD-10)	RR	10.2	Presence or absence of a nonpsychotic psychiatric disorder, below-normal social or intellectual functioning in adolescence, and socioeco-nomic status	7
Johnson and Breslau (31)	USA	Prospective	Wisconsin Longitudinal Study	4,858	53–54	53.6	Current smoking (habits and amount)	Major depression (DSM-IV)	OR	35.0	Sex and level of education	6
Pasco et al. (32)	Australia	Retrospective	The epidemiology of osteoporosis in Australian women,	671	20–84	0.0	Current smoking (>one or two cigarettes per day for at least 6 months)	Major depression (DSM-IV-TR)	OR	10.0	Socio-economic status, alcohol consumption, physical activity or physical illness	6
Sørensen et al. (33)	Denmark	Prospective	Copenhagen University Hospital	7,926	26	0.0	Current smoking (amount)	Schizophrenia, Bipolar disorder (ICD-8, ICD-10)	OR	40.0	Age, social class and psychopharmacological treatment at baseline	8
Goodwin et al. (34)	USA	Retrospective	Ohio Army National Guard Health Initiative	1,391	35	86.1	Current smoking, former smoking (habits)	Major depression (PHQ-9)	OR	3.0	Age and gender	6
Mojtabai et al. (35)	USA	Prospective	Residents of Hawaii and Alaska, sponsored by the National Institute on Alcohol Abuse and Alcoholism	33,154	> 18	47.1	Current smoking, former smoking (habits)	Major depression (DSM-IV)	OR	3.0	Gender, age, race/ethnicity, household income, education, marital status, physical illness, residence, region, lifetime history of other mental disorders, alcohol disorders, and nonalcohol drug disorders.	7
Wium-Andersen et al. (36)	Denmark	Prospective	Copenhagen General Population Study and the Copenhagen City Heat Study	63,296	> 20	NA	Current smoking (habits and amount)	Schizophrenia, major depression (ICD-8, ICD-10)	OR	6.0	Age, gender, alcohol consumption, physical activity, education, income, civil status, body mass index, plasma C-reactive protein and chronic disease	8
Kendler et al. (37)	Sweden	Prospective	Nationwide Swedish registers	1,647,728	27	14.2	Current smoking (amount)	Schizophrenia (ICD-9, ICD-10)	HR	18.5	Family socioeconomic status and community socioeconomic status	7
Chang et al. (38)	USA	Prospective	US female nurses	21,728	30–55	0.0	Current smoking, former smoking (amount)	Major depression (CESD-10, GDS-15)	HR	10.0	Age, education level, race, social network, body mass index, alternative Mediterranean diet score, moderate to vigorous activity, largest number of drinks in a single day, comorbidity, hours of actual sleep per day, and physical/functional limitation	8
Carroll et al. (39)	USA	Prospective	CARDIA	3,189	50	45.5	Current smoking (amount)	Major depression (CES-D)	OR	25.0	Sociodemographic, clinical, and behavioral covariates that are known risk factors for CAC and CVD: gender, race, age, education, total cholesterol, systolic blood pressure, diastolic blood pressure, body mass index, diabetes status, physical activity, alcohol use	7
Knight et al. (40)	USA	Prospective	Lupus Outcomes Study	546	18–45	6.8	Former smoking (habits)	Major depression (CES-D)	OR	12.0	Physical function, body mass index, diabetes mellitus, hypertension, and history of myocardial infarction or stroke	7
Zhang et al. (41)	Germany	Prospective	Large Dresden Predictor Study	1,196	18–25	0.0	Current smoking (habits and amount)	Major depression (DSM-IV)	OR	1.5	Body mass index, alcohol consumption, physical activity, and physical health	6
Liu et al. (42)	China	Prospective	SWHS and SMHS	103,595	40–74	48.3	Current smoking, former smoking (habits and amount)	Major depression (CES-D)	OR	11.0	Age, marriage, income, occupation and education	7
Tomita and Manuel (43)	South Africa	Retrospective	South African National Income Dynamics Study	14,118	NA	47.9	Current smoking (habits and amount)	Major depression (CES-D)	RR	7.0	Employment status, household income, and residence	6
Bach et al. (44)	Brazil	Prospective	Young adults living in the urban area of the city of Pelotas	1,244	18–24	44.8	Current smoking (habits)	Bipolar disorder (SDM-IV and ICD-10)	RR	5.0	Sex, age, education, socioeconomic, skin color, current work, liver with partner	7
King et al. (45)	UK	Retrospective	The Health Improvement Network primary care database	907,586	15–24	47.4	Current smoking (habits and amount)	Schizophrenia (ICD-9, ICD-10)	RR	2.0	Townsend deprivarion score	7
Bolstad et al. (46)	Finland	Prospective	The Northern Finland Birth Cohort 1986 Study	7,660	15/16	49.9	Current smoking (habits and amount)	Major depression, Bipolar disorder (ICD-10)	OR	17.0	Sex, parental psychiatric disorder, family structure, illicit substance use, Youth Self Report	8
Werneck et al. (47)	Brazil	Prospective	Non-probabilistic sample of Brazilian adults	4,725	18–59	81.0	Current smoking, former smoking (habits)	Major depression (BDI)	RR	3.1	Time between visits, chronological age, sex, number of metabolic risk factors, self-rated health, and HS-CRP	7
Fonseca et al. (48)	Brazil	Prospective	The Lifestyle and Health of University Students	1,034	16–25	57.1	Current smoking (habits)	Major depression (PHQ-9)	RR	3.0	Age, economic class, living situation, stress, alcohol consumption, sedentary behavior, perception of change in physical activity after entering the university, and short sleep duration	6
Zhao et al. (49)	China	Prospective	Nationally representative and annual longitudinal survey	16,892	> 18	52.1	Current smoking (habits)	Major depression (CESD-20)	RR	2.0	CESD-20 score of baseline survey, gender, marriage, education, area of residence, annual personal earning, social trust, self-rated health, morbidity of chronic disease, daytime nap duration, and drinking alcohol	6
Pengpid and Peltzer (50)	South Africa	Prospective	INDEPTH Community in South Africa	5,059	> 40	46.4	Current smoking (habits)	Major depression (CESD-20)	OR	4.0	Age, sex, country of birth, education, marital status, wealth index, alcohol dependence, physical activity, sedentary behavior, inadequate fruit/vegetable intake, body mass index, HIV positive, cardiovascular disease, functional disability	7
Kiviruusu et al. (51)	Finland	Prospective	Finnish-speaking pupils in Tampere	1,955	22–52	48.6	Current smoking (habits)	Major depression (BDI)	RR	30.0	Education, marital status, and alcohol use	7

### Major depression

**Figure 2:**
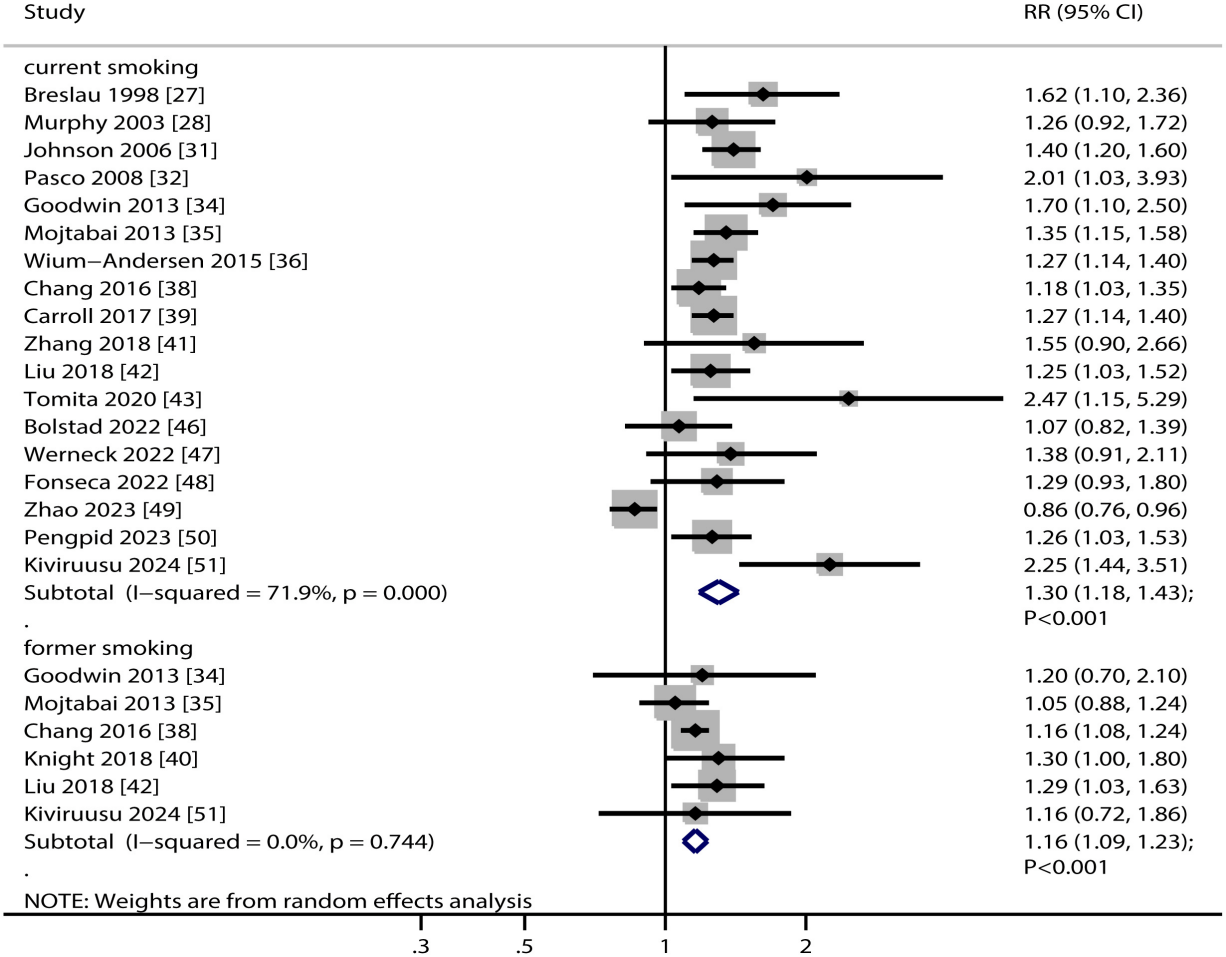
Association between smoking status and the risk of major depression. RR, relative risk; CI, confidence interval.

Eighteen studies reported an association between current smoking status and the risk of major depression. The overall analysis indicated that current smoking was associated with an increased risk of major depression (RR, 1.30; 95% CI, 1.18–1.43; *P* < 0.001; Figure 2). Significant heterogeneity was observed across the included studies (*I*^2^ = 71.9%, *P* < 0.001). Sensitivity analysis showed that the pooled RR with 95%CI was robust and not significantly altered by the sequential removal of any single study (Supplementary material 4). Subgroup analyses revealed that current smoking was associated with an increased risk of major depression in most subsets. However, no significant association was found between current smoking and the risk of major depression in the pooled male individuals. Additionally, the association between current smoking and the risk of major depression was influenced by the study design (*P* = 0.024) (Table 2). There was evidence of significant publication bias in the association between current smoking and the risk of major depression (*P* value for Egger's test, 0.041; *P* value for Begg's test, 0.037; Supplementary material 5). After adjusting for publication bias using the trim-and-fill method (52), the conclusions remained unchanged.

**Table 2:** Subgroup analyses for major psychiatric conditions.

**Outcomes**	**Factors**	**Subgroups**	**Number of studies**	**RR and 95%CI**	***P* value**	***I^2^* (%)**	***P* value for heterogeneity**	**Interaction test**
Major depression (current smoking vs non-smoking)	Study design	Prospective	15	1.28 (1.15–1.41)	< 0.001	73.3	< 0.001	0.024
		Retrospective	3	1.88 (1.37–2.59)	< 0.001	0.0	0.684	
	Gender	Male	3	1.60 (0.76–3.35)	0.213	85.9	0.001	0.688
		Female	6	1.37 (1.17–1.61)	< 0.001	21.1	0.274	
		Both	12	1.27 (1.13–1.44)	< 0.001	78.0	< 0.001	
	Smoking intensity^*^	Mild	4	1.66 (1.20–2.31)	0.002	82.0	< 0.001	0.115
		Moderate	5	1.26 (1.14–1.40)	< 0.001	0.0	0.596	
		Heavy	5	1.52 (1.37–1.68)	< 0.001	0.0	0.808	
	Follow-up	≥ 5.0 years	10	1.31 (1.19–1.45)	< 0.001	56.8	0.008	0.947
		< 5.0 years	8	1.30 (1.06–1.59)	0.011	79.0	< 0.001	
	Adjusted level	High	12	1.23 (1.10–1.38)	< 0.001	72.6	< 0.001	0.077
		Low	6	1.52 (1.24–1.87)	< 0.001	63.2	0.008	
	Study quality	High	10	1.29 (1.19–1.41)	< 0.001	48.5	0.030	0.645
		Low	8	1.37 (1.08–1.75)	0.010	81.7	< 0.001	
Major depression (former smoking vs non-smoking)	Study design	Prospective	5	1.16 (1.09–1.23)	< 0.001	0.0	0.432	0.905
		Retrospective	1	1.20 (0.69–2.08)	0.515	-	-	
	Gender	Male	2	1.34 (0.99–1.80)	0.058	8.0	0.297	0.362
		Female	3	1.16 (1.07–1.26)	< 0.001	2.2	0.360	
		Both	3	1.11 (0.97–1.29)	0.137	0.0	0.452	
	Follow-up	≥5.0 years	4	1.18 (1.10–1.25)	< 0.001	0.0	0.487	0.243
		< 5.0 years	2	1.06 (0.90–1.26)	0.468	0.0	0.649	
	Adjusted level	High	3	1.15 (1.08–1.22)	< 0.001	0.0	0.405	0.376
		Low	3	1.26 (1.04–1.53)	0.020	0.0	0.496	
	Study quality	High	5	1.16 (1.09–1.23)	< 0.001	0.0	0.432	0.905
		Low	1	1.20 (0.69–2.08)	0.515	-	-	
Schizophrenia (current smoking vs non-smoking)	Study design	Prospective	5	1.60 (1.01–2.52)	0.044	88.2	< 0.001	0.453
		Retrospective	1	2.26 (1.04–4.92)	0.040	-	-	
	Gender	Male	3	1.72 (0.60–4.96)	0.314	97.5	< 0.001	0.746
		Female	2	1.44 (1.19–1.76)	< 0.001	0.0	0.800	
		Both	2	2.35 (1.61–3.45)	< 0.001	27.7	0.240	
	Smoking intensity	Mild	6	1.54 (0.92–2.57)	0.100	86.5	< 0.001	0.443
		Moderate	6	1.65 (0.96–2.84)	0.069	90.5	< 0.001	
		Heavy	4	2.41 (1.08–5.36)	0.031	92.9	< 0.001	
	Follow-up	≥5.0 years	5	1.60 (1.01–2.52)	0.044	88.2	< 0.001	0.453
		< 5.0 years	1	2.26 (1.04–4.92)	0.040	-	-	
	Adjusted level	High	4	1.53 (0.90–2.59)	0.115	90.0	< 0.001	0.363
		Low	2	2.17 (1.27–3.71)	0.005	87.3	< 0.001	
	Study quality	High	6	1.78 (1.13–2.81)	0.012	93.1	< 0.001	-
		Low	0	-	-	-	-	

^*^Mild: 0–10 cigarettes; moderate: 10–20 cigarettes; heavy: ≥ 20 cigarettes.

Six studies reported an association between former smoking and the risk of major depression. The summary result indicated that former smoking was associated with an elevated risk of major depression (RR, 1.16; 95% CI, 1.09–1.23; *P* < 0.001; Figure 2). There was no evidence of heterogeneity among the included studies (*I*^2^ = 0.0%, *P* = 0.744). The pooled RR with 95%CI was stable and did not change after the exclusion of any single study (Supplementary material 4). Subgroup analyses revealed that former smoking was associated with an increased risk of major depression when combined with data from prospective cohort studies, among female participants, in studies with follow-ups ≥ 5.0 years, regardless of the degree of adjustment, and in high-quality study (Table 2). Reviewing the funnel plot could not rule out potential publication bias. However, the Egger (*P* = 0.632) and Begg (*P* = 0.707) tests indicated no significant publication bias (Supplementary material 5).

### Schizophrenia

**Figure 3:**
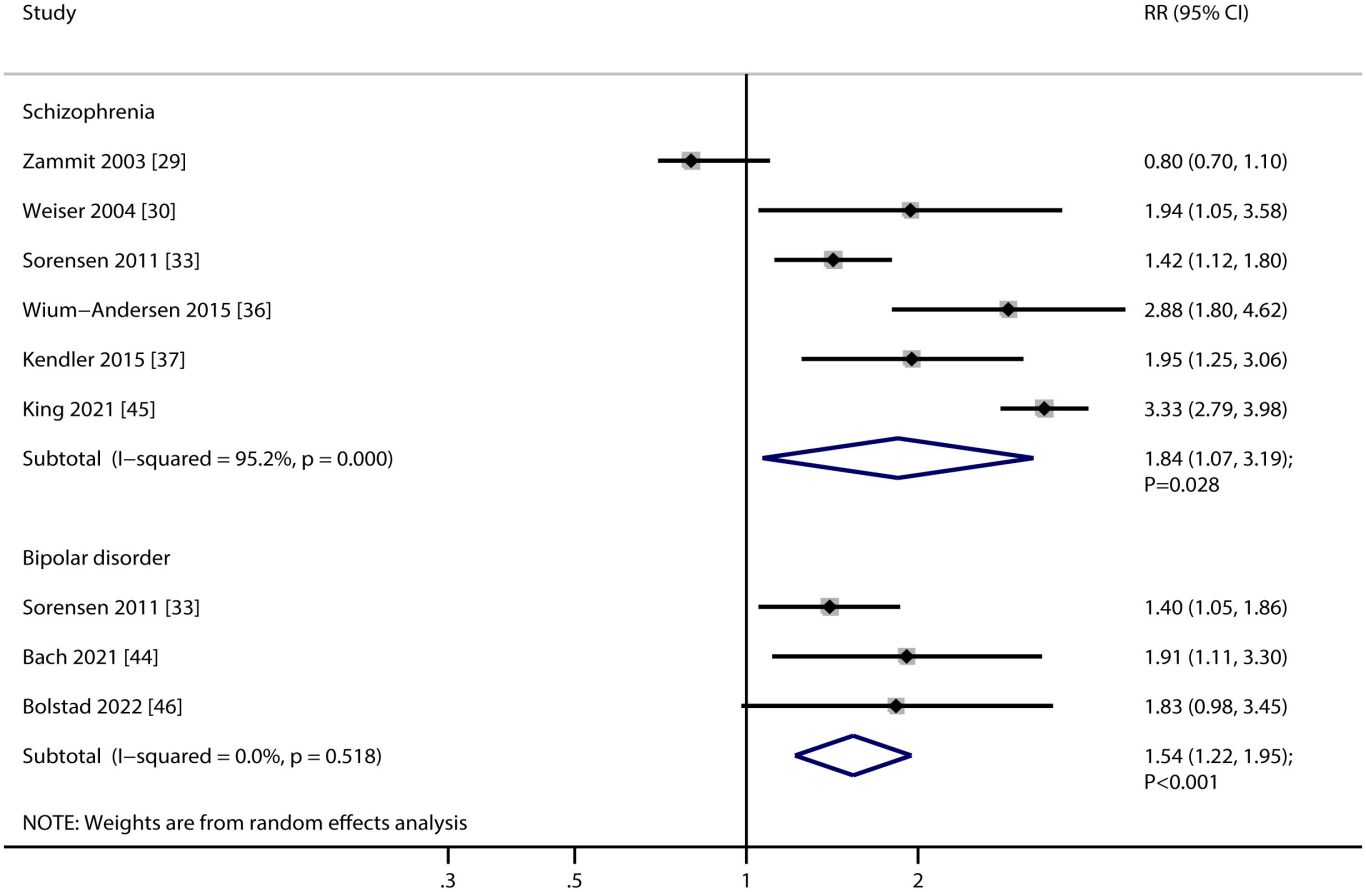
Association between current smoking and the risk of schizophrenia and bipolar disorder. RR, relative risk; CI, confidence interval.

Six studies reported an association between current smoking and the risk of schizophrenia. The pooled RR indicated that current smoking was associated with an increased risk of schizophrenia (RR, 1.84; 95% CI, 1.07–3.19; *P* = 0.028; Figure 3). Substantial heterogeneity was observed across the included studies (*I*^2^ = 95.2%, *P* < 0.001). Sensitivity analysis showed that the pooled RR with 95%CI varied due to the broad 95% CI (Supplementary material 4). Subgroup analyses found that current smoking was significantly related to an elevated risk of schizophrenia in the following subsets: irrespective of study design, pooled female individuals, or both male and female individuals, smoking intensity was heavy, irrespective of follow-up duration, studies with low adjusted levels, and studies with high quality (Table 2). There was no significant publication bias in the association between current smoking and the risk of schizophrenia (*P* value for Egger's test, 0.894; *P* value for Begg's test, 1.000; Supplementary material 5).

### Bipolar disorder

Three studies reported an association between current smoking and the risk of bipolar disorder. The pooled analysis indicated that current smoking was associated with an increased risk of bipolar disorder (RR, 1.54; 95% CI, 1.22–1.95; *P* < 0.001; Figure 3). No evidence of heterogeneity was observed among the included studies (*I*^2^ = 0.0%, *P* = 0.518). Due to the limited number of studies reporting this result, we did not perform sensitivity, subgroup, or publication bias analyses on the association between current smoking and the risk of bipolar disorder.

## Discussion

This extensive quantitative study encompassed 2,917,030 individuals across 25 cohort studies, representing a diverse range of participant characteristics. The results showed that current smoking was associated with an increased risk of major depression, schizophrenia, and bipolar disorder, while former smoking was linked to an elevated risk of major depression. Sensitivity analysis indicated that the pooled RR with 95% CI for the association between smoking status and major depression was robust. However, the association between current smoking and schizophrenia was not stable. Significant associations between smoking status and major psychiatric disorders were observed in most subgroups, and these associations might have been influenced by the study design.

This study found that current smoking is associated with an increased risk of major psychiatric disorders, including depression, schizophrenia, and bipolar disorder, these results were consistent with previous meta-analysis (14–16). The association between smoking and psychiatric disorders is complex and may be influenced by various factors, including individual differences, genetic predispositions, and environmental factors. For depression, nicotine, the primary component in cigarettes, rapidly reaches the brain upon inhalation. It binds to nicotinic acetylcholine receptors, triggering a cascade of events that ultimately lead to the release of neurotransmitters such as dopamine and serotonin. Chronic smoking can disrupt the normal regulation of these neurotransmitter systems (53). In the case of schizophrenia, current smoking may exacerbate the pathophysiological processes associated with the disorder. The dopamine hypothesis of schizophrenia posits that abnormal dopamine signaling in the brain is a key factor in the development of symptoms. Smoking-induced increases in dopamine release could potentially disrupt this already-abnormal dopamine regulation. Moreover, the neuroinflammatory response triggered by smoking can affect the integrity of the blood-brain barrier and lead to microglial activation. This inflammation may contribute to the neurodegenerative processes observed in schizophrenia. These structural and functional changes in the brain may be linked to the exacerbation of symptoms like hallucinations, delusions, and cognitive impairments in smokers with schizophrenia (54). Regarding bipolar disorder, the relationship with current smoking is also complex. Smoking-related oxidative stress can impact the brain's energy metabolism and mitochondrial function. Mitochondria play a crucial role in providing energy for neuronal activity, and any disruption in their function can lead to abnormal neuronal excitability. In bipolar disorder, mood swings between mania and depression may be influenced by these changes in neuronal energy metabolism (55). However, it is important to note that the analysis of the association between current smoking and bipolar disorder was based on only three studies, which represents a relatively small sample size. This limitation may potentially affect the reliability of the findings. Although all three studies were cohort studies with acceptable quality scores on the NOS, the small number of studies still restricts the generalizability of the results. Therefore, the findings regarding the association between smoking and bipolar disorder should be interpreted with caution. To enhance the robustness of these findings, future large - scale prospective studies are highly recommended to validate these results and further explore this relationship. Therefore, in-depth studies on the effects of smoking on psychiatric health remain an important area of research.

Similarly, we found that former smoking was associated with an increased risk of depression. Long-term smoking fosters the brain's dependence on nicotine-induced dopamine release. Upon quitting, dopamine levels drop significantly, impairing emotional regulation and increasing the risk of inducing or exacerbating depression. Accompanying withdrawal symptoms, such as anxiety, irritability, insomnia, and concentration difficulties, directly contribute to low mood, thereby enhancing the likelihood of depression. Additionally, the body continues to grapple with accumulated inflammation and oxidative stress from prior smoking, which persistently damage brain regions involved in emotional regulation (56). The absence of smoking as a coping mechanism, without the adoption of new healthy strategies, complicates emotional management, further escalating the risk of depression. However, the studies included did not provide evidence regarding the association between former smoking and the risk of schizophrenia and bipolar disorder, which necessitates further exploration in future research.

The results of the subgroup analyses were largely consistent with those of the overall analysis. Additionally, we observed that the association between smoking status and the risk of major psychiatric conditions can be influenced by the study design. Data in prospective studies are collected over time, allowing researchers to better control for confounding factors during the study process. This design helps in establishing a more robust causal relationship between smoking and psychiatric conditions. Conclusions from retrospective studies may be influenced by information and selection bias, as well as confounding factors. Researchers in retrospective studies cannot control the data collection process, which can lead to potential inaccuracies and biases.

Heterogeneity was observed in several parts of our meta-analysis, which is a common issue in such studies. There could be multiple sources contributing to this heterogeneity. Firstly, differences in study design might play a role. Some studies were prospective cohort studies, while others were retrospective, and this difference could lead to variations in data collection and follow-up methods, thereby affecting the observed associations. Secondly, population characteristics could be a significant source of heterogeneity. Variations in age, gender, and socioeconomic status among the participants of different studies might influence the relationship between smoking and psychiatric disorders. Additionally, differences in the definition of smoking status, such as what constitutes current smoking or former smoking, as well as varying cut-offs for smoking intensity across studies, may lead to inconsistent results. Geographical location and cultural differences among the study populations could also influence the results, as different regions may have different smoking patterns and cultural attitudes toward smoking and mental health. Our subgroup analyses have been instrumental in mitigating some of the heterogeneity issues. By dividing the studies into subgroups based on factors like gender, study design, and follow-up duration, we have been able to identify patterns and potential sources of variation. However, it is important to note that subgroup analyses have their limitations and may not fully explain all sources of heterogeneity. There could still be residual heterogeneity that we have not accounted for, possibly due to unmeasured factors or complex interactions between different variables. Future research could address these limitations by ensuring more detailed data collection, using standardized definitions for key variables, and employing more sophisticated statistical methods.

Our findings have important implications for public health. Given the observed associations between smoking and major psychiatric disorders, public health initiatives could be strengthened by integrating mental health screening into smoking cessation programs. Healthcare providers should consider screening individuals with psychiatric disorders for smoking behavior and vice versa. By identifying smokers at risk of developing psychiatric disorders early, targeted smoking cessation interventions could be implemented, potentially reducing the incidence of psychiatric disorders. Moreover, there could be significant economic implications. Early detection and intervention of smoking behavior among individuals with mental health conditions may lead to cost savings by reducing the burden on mental health services and minimizing the long-term healthcare costs associated with treating both smoking-related physical conditions and psychiatric disorders. In the clinical setting, our findings suggest that clinicians need to be more vigilant in assessing the smoking status of patients with psychiatric disorders. Smoking should be considered as a modifiable risk factor, and smoking cessation counseling and support should be incorporated into treatment plans. Collaborative care models, where mental health professionals and primary care physicians work together, could be beneficial. This interdisciplinary approach could improve the management of comorbid conditions, as addressing both smoking and psychiatric disorders simultaneously may lead to better patient outcomes. Furthermore, education and prevention play a crucial role. Public education about the link between smoking and psychiatric disorders could change behaviors and attitudes toward smoking, particularly among vulnerable populations. Educational campaigns in schools, workplaces, and healthcare settings should target both smokers and those at risk of developing psychiatric disorders. By raising awareness, individuals may be more motivated to quit smoking or avoid starting, thereby reducing the risk of developing psychiatric disorders.

Our study has three key strengths that are worth emphasizing. First, we included only cohort studies, which can, to some extent, verify the causal association between smoking and psychiatric disorders. This design allows for a more robust assessment of temporal relationships and reduces the risk of reverse causality. Second, the large sample size in our study enables us to quantitatively assess the association between smoking and the risk of psychiatric disorders. This increases the statistical power and potentially renders our findings more reliable compared to those from individual studies. Third, we conducted an exploratory analysis to evaluate the strength of the association between smoking and the risk of psychiatric disorders in specific populations. This provides valuable insights into how these associations may vary across different subgroups, enhancing the depth and applicability of our findings.

The limitations of this study include the following: (1) due to the observational nature of the included studies, we cannot establish causation. Observational studies, such as the cohort studies included in our meta-analysis, are inherently subject to residual confounding. Residual confounding occurs when there are unmeasured or inadequately measured factors that could influence the relationship between smoking and major psychiatric disorders; (2) different studies included different adjustment factors, which may play important roles in the occurrence and development of psychiatric disorders. This inconsistency can affect the comparability and reliability of the findings; (3) the established causal relationship between smoking and the development of major psychiatric disorders appears limited and necessitates additional validation (57); (4) regarding the analysis of socio-economic status, we recognize its potential importance in understanding the relationship between smoking and psychiatric disorders. However, due to the lack of reporting of this information in most of the included studies, we were unable to perform a subgroup analysis based on this variable; (5) this study was based on published articles, and access to unpublished data was unavailable. This makes publication bias an unavoidable issue, potentially leading to an overestimation or underestimation of the true effect sizes; and (6) the analysis was based on aggregated data (without individual data), which limits our ability to perform more detailed correlation analyses and obtain comprehensive results.

## Conclusion

Current smoking is associated with an elevated risk of several major psychiatric conditions, including depression, schizophrenia, and bipolar disorder. Additionally, former smokers exhibit an increased vulnerability to severe depression. The association between smoking status and the risk of major psychiatric disorders may be influenced by the study design, which suggests that clinicians and mental health professionals should incorporate patients' smoking history and quitting status into their assessment and management of psychiatric disorders, employing targeted interventions accordingly. For individuals who have quit smoking, intensified psychological support and social interventions may be particularly crucial to prevent or alleviate potential psychiatric symptoms. In the future, more studies are needed to further elucidate the mechanisms underlying the association between smoking and psychiatric disorders. This could involve more comprehensive investigations of biological pathways and the role of confounding factors. Moreover, to establish a more definitive causal relationship, future research should consider employing methods like Mendelian randomization or advanced causal inference techniques.

## Data Availability

The original contributions presented in the study are included in the article/Supplementary material, further inquiries can be directed to the corresponding author.
