# Supplementary material for: Association between cigarette smoking and the risk of major psychiatric disorders: a systematic review and meta-analysis in depression, schizophrenia, and bipolar disorder
Source: Front Med (Lausanne). 2025 Feb 13;12:1529191. doi: 10.3389/fmed.2025.1529191 (PMC11865063; doi:10.3389/fmed.2025.1529191)
Supplement: Supplementary file 1 [file Data_Sheet_1.docx]

Table S1. Quality scores of cohort studies using Newcastle-Ottawa Scale.

| Study | Selection | | | | Comparability | Outcome | | | NOS |
| --- | --- | --- | --- | --- | --- | --- | --- | --- | --- |
|  | Representativeness of the exposed cohort | Selection of the non exposed cohort | Ascertainment  of smoking | Demonstration that outcomes was not present at start of study | Comparability on the basis of the design or analysis | Assessment of outcome | Adequate follow-up duration | Adequate follow-up rate | Overall score |
| Breslau 1998 [27] | 1 | 1 | 1 | 1 | 2 | 1 | 0 | 1 | 8 |
| Murphy 2003 [28] | 0 | 1 | 1 | 1 | 1 | 1 | 1 | 0 | 6 |
| Zammit 2003 [29] | 1 | 1 | 1 | 1 | 1 | 1 | 1 | 0 | 7 |
| Weiser 2004 [30] | 1 | 1 | 1 | 1 | 1 | 1 | 1 | 0 | 7 |
| Johnson 2006 [31] | 0 | 1 | 1 | 1 | 1 | 1 | 1 | 0 | 6 |
| Pasco 2008 [32] | 0 | 0 | 1 | 1 | 1 | 1 | 1 | 1 | 6 |
| Sørensen 2011 [33] | 1 | 1 | 1 | 1 | 2 | 1 | 1 | 0 | 8 |
| Goodwin 2013 [34] | 0 | 0 | 1 | 1 | 2 | 1 | 0 | 1 | 6 |
| Mojtabai 2013 [35] | 1 | 1 | 1 | 1 | 1 | 1 | 0 | 1 | 7 |
| Wium-Andersen 2015 [36] | 1 | 1 | 1 | 1 | 2 | 1 | 0 | 1 | 8 |
| Kendler 2015 [37] | 1 | 1 | 1 | 1 | 1 | 1 | 1 | 0 | 7 |
| Chang 2016 [38] | 1 | 1 | 1 | 1 | 1 | 1 | 1 | 1 | 8 |
| Carroll 2017 [39] | 0 | 1 | 1 | 1 | 1 | 1 | 1 | 1 | 7 |
| Knight 2018 [40] | 0 | 1 | 1 | 1 | 1 | 1 | 1 | 1 | 7 |
| Zhang 2018 [41] | 0 | 1 | 1 | 1 | 1 | 1 | 0 | 1 | 6 |
| Liu 2018 [42] | 1 | 0 | 1 | 1 | 1 | 1 | 1 | 1 | 7 |
| Tomita 2020 [43] | 1 | 0 | 1 | 1 | 1 | 1 | 0 | 1 | 6 |
| Bach 2021 [44] | 0 | 1 | 1 | 1 | 2 | 1 | 0 | 1 | 7 |
| King 2021 [45] | 1 | 1 | 1 | 1 | 1 | 1 | 0 | 1 | 7 |
| Bolstad 2022 [46] | 1 | 1 | 1 | 1 | 1 | 1 | 1 | 1 | 8 |
| Werneck 2022 [47] | 1 | 1 | 1 | 1 | 1 | 1 | 0 | 1 | 7 |
| Fonseca 2022 [48] | 0 | 1 | 1 | 1 | 1 | 1 | 0 | 1 | 6 |
| Zhao 2023 [49] | 1 | 0 | 1 | 1 | 1 | 1 | 0 | 1 | 6 |
| Pengpid 2023 [50] | 1 | 1 | 1 | 1 | 1 | 1 | 0 | 1 | 7 |
| Kiviruusu 2024 [51] | 0 | 1 | 1 | 1 | 1 | 1 | 1 | 1 | 7 |
