# Supplementary material for: Association between cigarette smoking and the risk of major psychiatric disorders: a systematic review and meta-analysis in depression, schizophrenia, and bipolar disorder
Source: Front Med (Lausanne). 2025 Feb 13;12:1529191. doi: 10.3389/fmed.2025.1529191 (PMC11865063; doi:10.3389/fmed.2025.1529191)
Supplement: Supplementary file 3 [file Data_Sheet_3.docx]

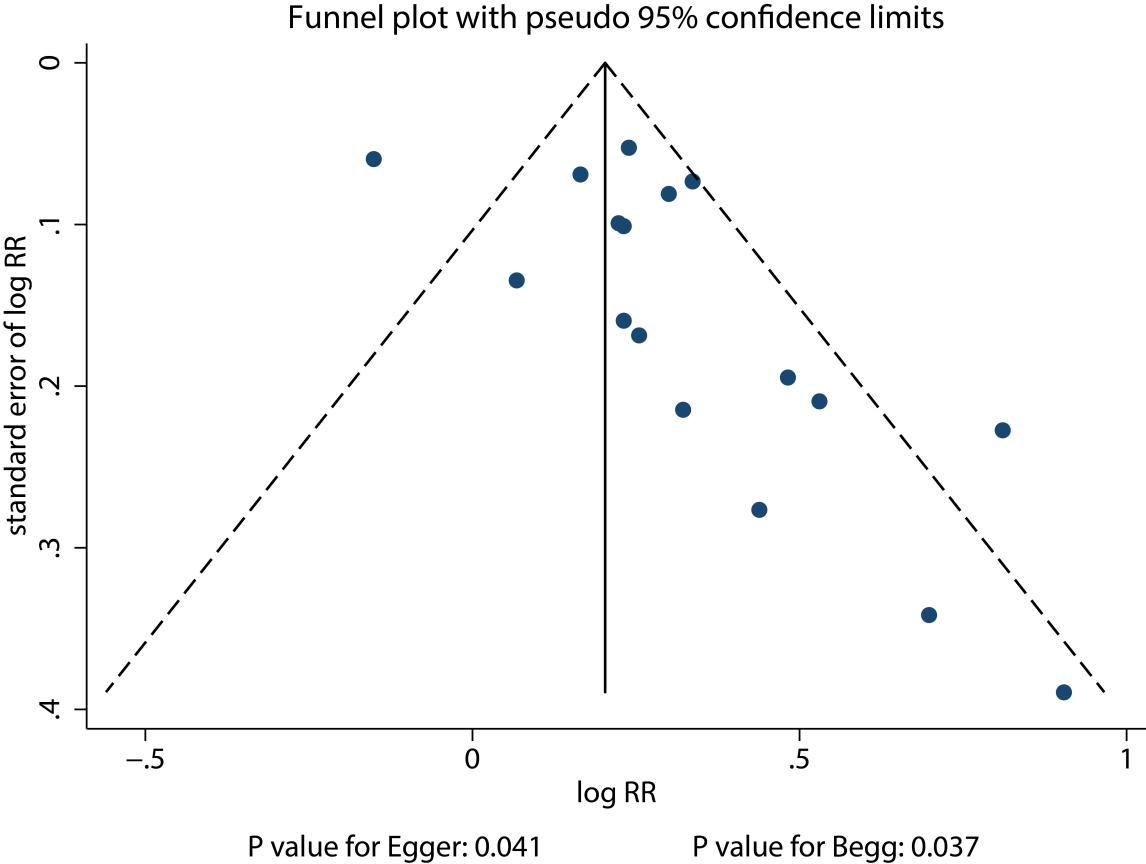


Figure S1. Funnel plot for the relation between current smoking and major depression risk


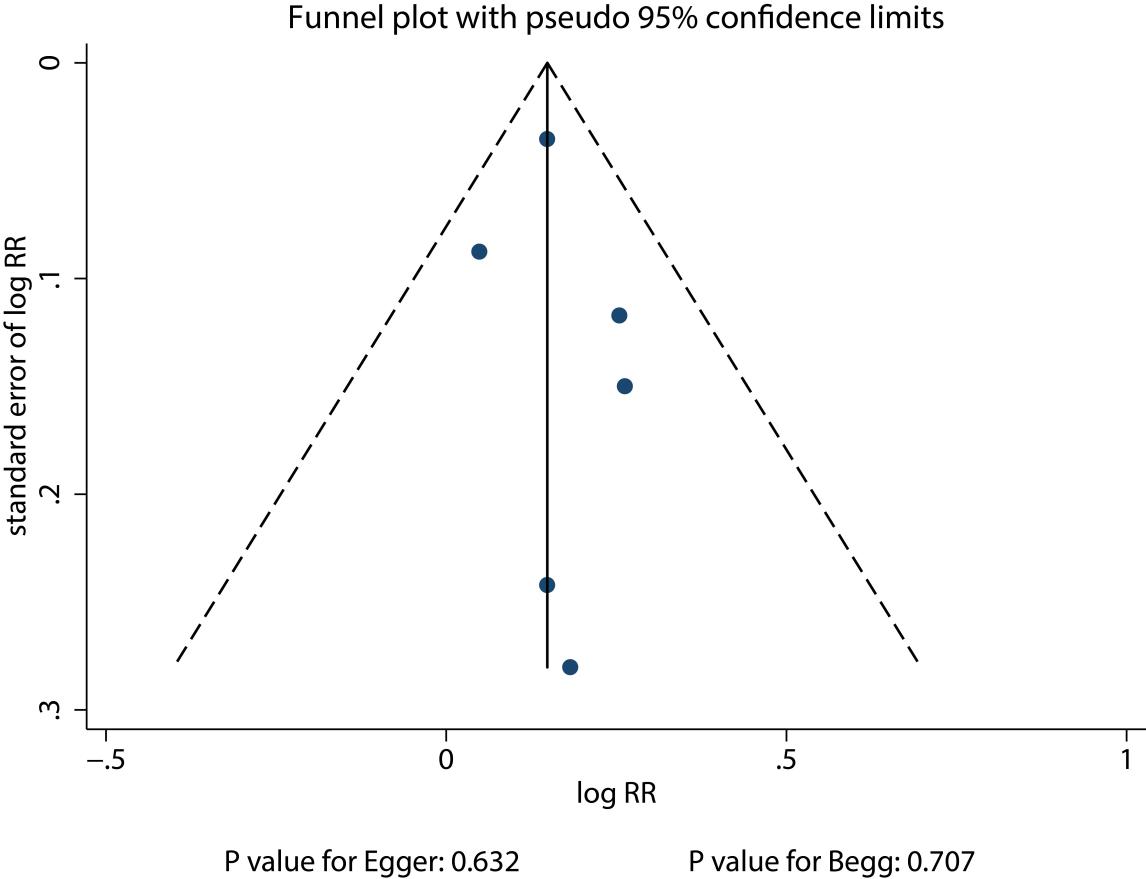


Figure S2. Funnel plot for the relation between former smoking and major depression risk


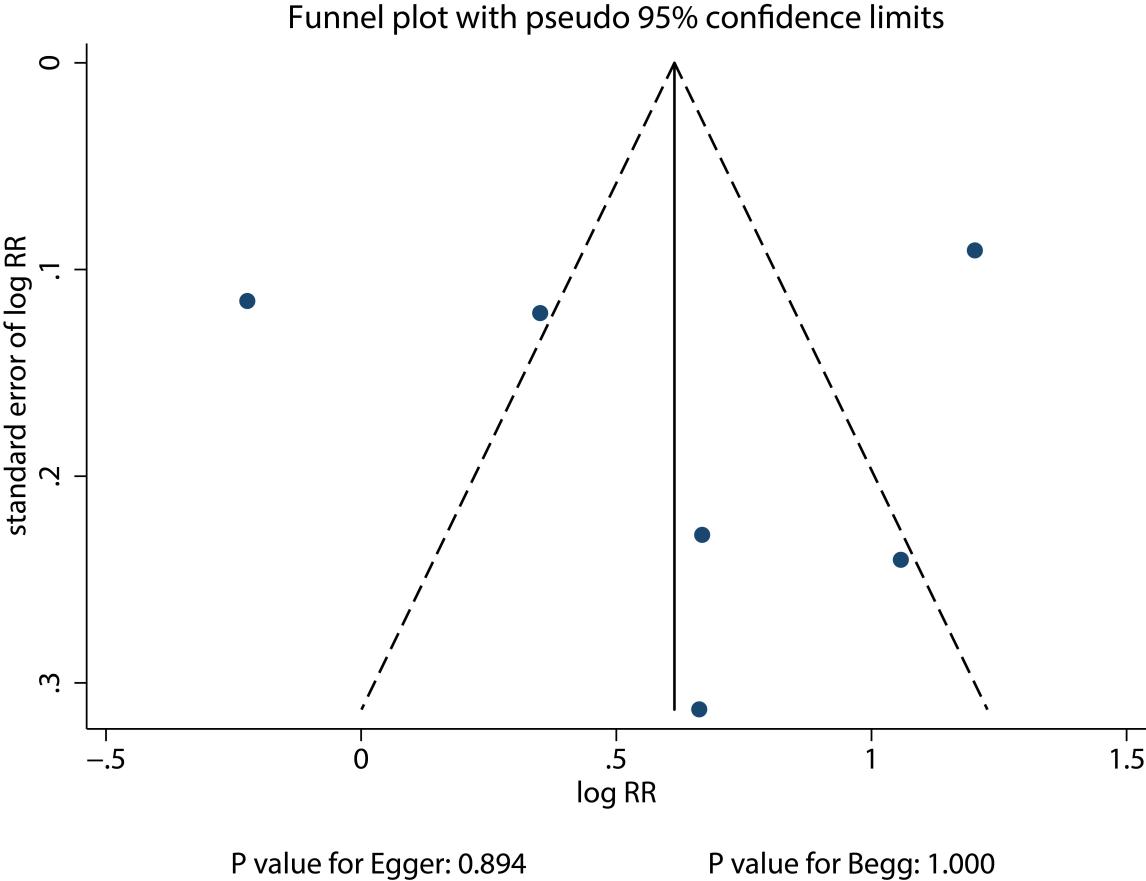


Figure S3. Funnel plot for the relation between current smoking and schizophrenia risk
