# Supplementary material for: Association between cigarette smoking and the risk of major psychiatric disorders: a systematic review and meta-analysis in depression, schizophrenia, and bipolar disorder
Source: Front Med (Lausanne). 2025 Feb 13;12:1529191. doi: 10.3389/fmed.2025.1529191 (PMC11865063; doi:10.3389/fmed.2025.1529191)
Supplement: Supplementary file 4 [file Table_1.docx]

**Search strategy**

[(MH "Smoking") OR (MH "Tobacco") OR (MH "Tobacco Use Cessation") OR (MH "Smoking Cessation") OR (MH "Tobacco Use Disorder") OR (MH "Nicotine") OR TI smoking OR TI cigarette* OR TI nicotine OR TI tobacco] AND [(MH "Mental Disorders") OR (MH "Anxiety Disorders+") OR (MH "Mood Disorders+") OR (MH "Schizophrenia and Disorders with Psychotic Features+") OR (MH "Personality Disorders+") OR TI mental OR TI psychiatric OR TI psychosis OR TI psychotic OR TI schiz* OR TI mood disorder* OR TI affective disorder* OR TI bipolar OR TI depression OR TI depressive disorder* OR TI personality disorder*].
