# Supplementary material for: Association between cigarette smoking and the risk of major psychiatric disorders: a systematic review and meta-analysis in depression, schizophrenia, and bipolar disorder
Source: Front Med (Lausanne). 2025 Feb 13;12:1529191. doi: 10.3389/fmed.2025.1529191 (PMC11865063; doi:10.3389/fmed.2025.1529191)
Supplement: Supplementary file 5 [file Table_2.docx]

**Excluding studies**

***Not cohort design***

1. Burlaka J, Johnson RM, Marsack-Topolewski CN, Hughesdon K, Owczarzak J, Serdiuk O, Bogdanov R, Burlaka V. Association between Current Substance Use, Healthy Behaviors, and Depression among Ukrainian College Students. Int J Environ Res Public Health. 2024 May 2;21(5):586. doi: 10.3390/ijerph21050586.
2. Hong C, Mammadli T, Lunchenkov N, Garner A, Howell S, Holloway IW. Changes in alcohol, tobacco, cannabis, and other substance use and its association with mental health during the COVID-19 pandemic among sexual minority men in Eastern European and Central Asian countries. J Affect Disord. 2024 Aug 15;359:302-307. doi: 10.1016/j.jad.2024.05.089.
3. George AB, Gupta A, Jain R, Sood M, Sarkar S. Serum brain-derived neurotrophic factor level and its relation with cannabis use disorder and schizophrenia: A cross-sectional exploratory study in patients at a tertiary care hospital. Indian J Pharmacol. 2024 Mar 1;56(2):91-96. doi: 10.4103/ijp.ijp_771_22.
4. Jacovides C, Papadopoulou SK, Pavlidou E, Dakanalis A, Alexatou O, Vorvolakos T, Lechouritis E, Papacosta E, Chrysafi M, Mitsiou M, Mentzelou M, Kosti RI, Giaginis C. Association of Pregnant Women's Perinatal Depression with Sociodemographic, Anthropometric and Lifestyle Factors and Perinatal and Postnatal Outcomes: A Cross-Sectional Study. J Clin Med. 2024 Apr 3;13(7):2096. doi: 10.3390/jcm13072096.
5. Millar SR, Harrington JM, Perry IJ, Phillips CM. Lifestyle factors and BMI attenuate relationships between biomarkers of inflammation and depressive symptoms and well-being: A cross-sectional study. Brain Behav Immun Health. 2024 Mar 18;37:100759. doi: 10.1016/j.bbih.2024.100759.
6. Aghasizadeh Sherbaf R, Kaposvári GM, Nagy K, Álmos ZP, Baráth Z, Matusovits D. Oral Health Status and Factors Related to Oral Health in Patients with Schizophrenia: A Matched Case-Control Observational Study. J Clin Med. 2024 Mar 10;13(6):1584. doi: 10.3390/jcm13061584.
7. Reynales-Shigematsu LM, Rivera-Rivera L, Séris-Martínez M, Saenz-de-Miera B. A Cross-Sectional Analysis of Parental Behavior and Adolescent Mental Health in Mexico: Insights into Excessive Alcohol Intake, Tobacco Use, Suicidal Behavior, and Depressive Symptomatology. Healthcare (Basel). 2024 Mar 13;12(6):641. doi: 10.3390/healthcare12060641.
8. Pool M, Sorsdahl K, Myers B, van der Westhuizen C. The prevalence of and factors associated with depressive and anxiety symptoms during the COVID-19 pandemic among healthcare workers in South Africa. PLoS One. 2024 Mar 7;19(3):e0299584.
9. Zvolensky MJ, Shepherd JM, Argueta S, Bizier A, Clausen BK, Buckner JD, de Dios MA, Cano MÁ. Evaluating the indirect roles of anxiety and depressive symptoms in the relations between negative emotional reactivity to racial/ethnic stress and cigarette smoking among Hispanic adults who smoke. Exp Clin Psychopharmacol. 2024 Mar 7. doi: 10.1037/pha0000708.
10. Mo H, Wang C, Li Y. Recent tobacco smoking, restaurant and in-car secondhand smoke exposure are associated with depressive symptoms among young adults: a population-based cross-sectional analysis. Sci Rep. 2024 Mar 4;14(1):5290. doi: 10.1038/s41598-024-54575-y.
11. Muyiduli X, Zhang R, Zhang J, Zhe W, Dong Y, Wang W, Fang P, Zhang Y, Zhang S, Sulidan A, Rejiafu S, Sun J. Prevalence and relevant factors of depression among adolescents in Xinjiang, China: A cross-sectional survey. Medicine (Baltimore). 2024 Feb 16;103(7):e37090. doi: 10.1097/MD.0000000000037090.
12. Yeo PME, Qin VM, Ang CS, Chia M, Ho RM, Ho AHY, Car J. Prevalence and correlates of depressive symptoms among matriculated university students in Singapore during Covid-19 pandemic: findings from a repeated cross-sectional analysis. BMC Public Health. 2024 Feb 13;24(1):454. doi: 10.1186/s12889-024-17866-7.
13. Jaka S, Singh S, Vashist S, Pokhrel S, Saldana E, Sejdiu A, Taneja S, Arisoyin A, Mogallapu R, Gunturu S, Bachu A, Patel RS. Pediatric anti-N-methyl-D-aspartate receptor (NMDAR) encephalitis: Exploring psychosis, related risk factors, and hospital outcomes in a nationwide inpatient sample: A cross-sectional study. PLoS One. 2024 Feb 13;19(2):e0296870. doi: 10.1371/journal.pone.0296870.
14. Jaka S, Singh S, Vashist S, Pokhrel S, Saldana E, Sejdiu A, Taneja S, Arisoyin A, Mogallapu R, Gunturu S, Bachu A, Patel RS. Pediatric anti-N-methyl-D-aspartate receptor (NMDAR) encephalitis: Exploring psychosis, related risk factors, and hospital outcomes in a nationwide inpatient sample: A cross-sectional study. PLoS One. 2024 Feb 13;19(2):e0296870. doi: 10.1371/journal.pone.0296870.
15. Li L, Yang P, Duan Y, Xie J, Liu M, Zhou Y, Luo X, Zhang C, Li Y, Wang J, Chen Z, Zhang X, Cheng ASK. Association between dietary diversity, sedentary time outside of work and depressive symptoms among knowledge workers: a multi-center cross-sectional study. BMC Public Health. 2024 Jan 2;24(1):53. doi: 10.1186/s12889-023-17567-7.
16. Kurt ZK, Demir Hacıosmanoğlu G, Yıldırım M, Özaslan A. Adolescent smoking patterns: Associations with sociodemographic factors, cyberbullying, and psychiatric diagnoses in an outpatient clinical sample. J Ethn Subst Abuse. 2024 Jan 2:1-19. doi: 10.1080/15332640.2023.2299873.
17. Ma G, Tian Y, Zi J, Hu Y, Li H, Zeng Y, Luo H, Xiong J. Systemic inflammation mediates the association between environmental tobacco smoke and depressive symptoms: A cross-sectional study of NHANES 2009-2018. J Affect Disord. 2024 Mar 1;348:152-159. doi: 10.1016/j.jad.2023.12.060.
18. Mahmood N, Goldstein S, Thiele A, Trotchie M, des Bordes J. Smoking and Depression Risk Reduction in a Primary Care Setting. J Prim Care Community Health. 2023 Jan-Dec;14:21501319231213748. doi: 10.1177/21501319231213748.
19. Kuang Z, Zhang B, Li X, Zhao J, Xu J, Wei Z, Li L, Dong J, Yu X, Li J, Zhao J, Shi B. Evaluation of lifestyle behaviors, anxiety and depression in patients with hematologic disorders. Medicine (Baltimore). 2023 Nov 17;102(46):e35863. doi: 10.1097/MD.0000000000035863.
20. Fu C, Cao L, Yang F. Prevalence and determinants of depressive symptoms among community-dwelling older adults in China based on differences in living arrangements: a cross-sectional study. BMC Geriatr. 2023 Oct 10;23(1):640. doi: 10.1186/s12877-023-04339-6.
21. Jumani S, Osoble A, Ahmed T, Rastegarlari T, Hassan M, Sreedharan J. Prevalence of Depressive Symptoms Among an Undergraduate Health Sciences Student Population: A Cross-Sectional Study. Cureus. 2023 Aug 8;15(8):e43117. doi: 10.7759/cureus.43117.
22. Floyd J, Mallow J, Wang K, Davis SM, Carpenter R, Theeke L. Differences in smoking behaviors and readiness to change for patients with COPD and differing categories of depressive symptoms: a descriptive cross-sectional design. BMC Pulm Med. 2023 Sep 8;23(1):335. doi: 10.1186/s12890-023-02621-2.
23. Trani JF, Yen BJ, Duncan A, Bakhshi P, Palmo T, Jadhav S, Deshpande S. People with mental illness stigmatize mental illness less: A comparison study between a hospital-based sample of people with mental illness and a non-clinical general population sample in urban India. Transcult Psychiatry. 2023 Dec;60(6):954-972. doi: 10.1177/13634615231179265.
24. Çelik DÖ, Haney MÖ. The relationship between depression, healthy lifestyle behaviors and internet addiction: a cross-sectional study of the athlete university students in Turkey. Front Psychiatry. 2023 Jul 7;14:1222931. doi: 10.3389/fpsyt.2023.1222931.
25. Wang M, Mou X, Li T, Zhang Y, Xie Y, Tao S, Wan Y, Tao F, Wu X. Association Between Comorbid Anxiety and Depression and Health Risk Behaviors Among Chinese Adolescents: Cross-Sectional Questionnaire Study. JMIR Public Health Surveill. 2023 Jul 5;9:e46289. doi: 10.2196/46289.
26. Leung DYP, Leung SF, Zhang XL, Ruan JY, Yeung WF, Mak YW. Factors associated with severe depressive symptoms among Chinese secondary school students in Hong Kong: a large cross-sectional survey. Front Public Health. 2023 Jun 6;11:1148528. doi: 10.3389/fpubh.2023.1148528.
27. Gravely S, Driezen P, McClure EA, Smith DM, Fong GT. Prevalence of depressive symptoms and cannabis use among adult cigarette smokers in Canada: cross-sectional findings from the 2020 International Tobacco Control Policy Evaluation Project Canada Smoking and Vaping Survey. CMAJ Open. 2023 Jun 13;11(3):E516-E526. doi: 10.9778/cmajo.20220081.
28. Ren Z, Du Y, Lian X, Sun J, Zheng X, Liu J. The dilution effects of healthy lifestyles on the risk of depressive symptoms attributed to life-course disadvantages among Chinese middle-aged and older adults. J Affect Disord. 2023 Sep 1;336:97-105. doi: 10.1016/j.jad.2023.05.064.
29. Asrullah M, L'Hoir M, Paulo MJ, Feskens EJM, Melse-Boonstra A. Determinants of Common Mental Disorders (CMD) among adolescent girls aged 15-19 years in Indonesia: Analysis of the 2018 National Basic Health Survey Data. PLOS Glob Public Health. 2022 Mar 15;2(3):e0000232. doi: 10.1371/journal.pgph.0000232.
30. Mongan D, Raj Susai S, Föcking M, Byrne JF, Zammit S, Cannon M, Cotter DR. Associations between plasma inflammatory markers and psychotic disorder, depressive disorder and generalised anxiety disorder in early adulthood: A nested case-control study. Brain Behav Immun. 2023 Jul;111:90-100. doi: 10.1016/j.bbi.2023.03.025.
31. Du H, Yang B, Wang H, Zeng Y, Xin J, Li X. The non-linear correlation between the volume of cerebral white matter lesions and incidence of bipolar disorder: A secondary analysis of data from a cross-sectional study. Front Psychiatry. 2023 Mar 16;14:1149663. doi: 10.3389/fpsyt.2023.1149663.
32. Zhang X, Zhang L, Liu Y, Lin Y, Yang X, Gong L, Chang C. The relationship between unhealthy lifestyle patterns and depressive symptoms among residents in Beijing, China: A community-based cross-sectional study. Front Public Health. 2023 Apr 12;11:1055209. doi: 10.3389/fpubh.2023.1055209.
33. Sánchez-Gutiérrez T, Rodríguez-Toscano E, Roldán L, Ferraro L, Parellada M, Calvo A, López G, Rapado-Castro M, La Barbera D, La Cascia C, Tripoli G, Di Forti M, Murray RM, Quattrone D, Morgan C, van Os J, García-Portilla P, Al-Halabí S, Bobes J, de Haan L, Bernardo M, Santos JL, Sanjuán J, Arrojo M, Ferchiou A, Szoke A, Rutten BP, Stilo S, D'Andrea G, Tarricone I; EU-GEI WP2 Group; Díaz-Caneja CM, Arango C. Tobacco use in first-episode psychosis, a multinational EU-GEI study. Psychol Med. 2023 Nov;53(15):7265-7276. doi: 10.1017/S0033291723000806.
34. Wang D, Ma Z, Fan Y, Chen H, Sun M, Fan F. Tobacco smoking, second-hand smoking exposure in relation to psychotic-like experiences in adolescents. Early Interv Psychiatry. 2024 Feb;18(2):102-112. doi: 10.1111/eip.13439.
35. Idris H, Tuzzahra F. Factors associated with depressive symptoms among adolescents in Indonesia: A cross-sectional study of results from the Indonesia Family Life Survey. Malays Fam Physician. 2023 Mar 11;18:29. doi: 10.51866/oa.265.
36. Sadarangani KP, Schuch FB, De Roia G, Martínez-Gomez D, Chávez R, Lobo P, Cristi-Montero C, Werneck AO, Alzahrani H, Ferrari G, Ibanez A, Silva DR, Von Oetinger A, Matias TS, Grabovac I, Meyer J. Exchanging screen for non-screen sitting time or physical activity might attenuate depression and anxiety: A cross-sectional isotemporal analysis during early pandemics in South America. J Sci Med Sport. 2023 Jun;26(6):309-315. doi: 10.1016/j.jsams.2023.04.007.
37. You MA, Choi J, Son YJ. Associations of dual use of tobacco cigarettes and e-cigarettes, sleep duration, physical activity and depressive symptoms among middle-aged and older Korean adults. Nurs Open. 2023 Jun;10(6):4071-4082. doi: 10.1002/nop2.1667.
38. Kassew T, Tilahun SY, Alemayehu BF, Getnet B, Demilew D, Tarekegn GE, Alemu K, Yesuf YM, Oumer M, Mehari EA, Melkam M, Nenko G. Psychotic symptoms and its associated factors relating to psychoactive substance use among the youth population in Northwest Ethiopia. Front Psychiatry. 2023 May 5;14:1045111. doi: 10.3389/fpsyt.2023.1045111.
39. Thorisdottir IE, Agustsson G, Oskarsdottir SY, Kristjansson AL, Asgeirsdottir BB, Sigfusdottir ID, Valdimarsdottir HB, Allegrante JP, Halldorsdottir T. Effect of the COVID-19 pandemic on adolescent mental health and substance use up to March, 2022, in Iceland: a repeated, cross-sectional, population-based study. Lancet Child Adolesc Health. 2023 May;7(5):347-357. doi: 10.1016/S2352-4642(23)00022-6.
40. Jafarzadeh NS, Bello MS, Wong M, Cho J, Leventhal AM. Associations between anxiety symptoms and barriers to smoking cessation among African Americans who smoke cigarettes daily. Drug Alcohol Depend. 2023 Apr 1;245:109808. doi: 10.1016/j.drugalcdep.2023.109808.
41. Fukushima S, Suzuki F, Tsujiguchi H, Hara A, Miyagi S, Kannon T, Suzuki K, Shimizu Y, Nguyen TTT, Yanagisawa T, Oku F, Sato K, Nakamura M, Hayashi K, Shibata A, Konoshita T, Kambayashi Y, Tsuboi H, Tajima A, Nakamura H. Relationships among Depressive Symptoms, Body Weight, and Chronic Pain: A Cross-Sectional Analysis of the Shika Study. Behav Sci (Basel). 2023 Jan 20;13(2):86. doi: 10.3390/bs13020086.
42. Wu Z, Yue Q, Zhao Z, Wen J, Tang L, Zhong Z, Yang J, Yuan Y, Zhang X. A cross-sectional study of smoking and depression among US adults: NHANES (2005-2018). Front Public Health. 2023 Jan 30;11:1081706. doi: 10.3389/fpubh.2023.1081706.
43. Provenzani U, De Micheli A, Damiani S, Oliver D, Brondino N, Fusar-Poli P. Physical Health in Clinical High Risk for Psychosis Individuals: A Cross-Sectional Study. Brain Sci. 2023 Jan 12;13(1):128. doi: 10.3390/brainsci13010128.
44. Singh S, Lai CH, Iderus NHM, Ghazali SM, Ahmad LCRQ, Cheng LM, Nadzri MN, Zulkifli AA, Suppiah J, Zaki RA, Farid NDN, Supramanian RK, Nordin AA, Lin CZ, Kaur K, Ghazali NM. Prevalence and Determinants of Depressive Symptoms among Young Adolescents in Malaysia: A Cross-Sectional Study. Children (Basel). 2023 Jan 11;10(1):141. doi: 10.3390/children10010141.
45. Cyran A, Piotrowski P, Samochowiec J, Grąźlewski T, Misiak B. Risk factors of deficit and non-deficit schizophrenia: Results from a cross-sectional study. Rev Psiquiatr Salud Ment (Engl Ed). 2022 Oct-Dec;15(4):223-229. doi: 10.1016/j.rpsmen.2022.05.001.
46. Kong JY, Hong H, Kang H. Relationship between physical activity and depressive symptoms in older Korean adults: moderation analysis of muscular strength. BMC Geriatr. 2022 Nov 21;22(1):884. doi: 10.1186/s12877-022-03610-6.
47. Raffetti E, Donato F, Triolo F, Andersson F, Forsell Y, Galanti MR. Country differences in the cross-sectional associations between smoking and depressive symptoms in adolescence. Eur J Public Health. 2022 Nov 29;32(6):913-918. doi: 10.1093/eurpub/ckac155.
48. Zong Q, Li H, Jiang N, Gong Y, Zheng J, Yin X. Prevalence and determinants of smoking behavior among physicians in emergency department: A national cross-sectional study in China. Front Public Health. 2022 Oct 17;10:980208. doi: 10.3389/fpubh.2022.980208.
49. Jones-Patten A, Wang Q, Molebatsi K, Novotny TE, Siddiqi K, Modongo C, Zetola NM, Mbongwe B, Shin SS. Depression, Anxiety, and Cigarette Smoking Among Patients with Tuberculosis. Clin Nurs Res. 2023 Jan;32(1):22-28. doi: 10.1177/10547738221132096.
50. Liu Q, Leng P, Gu Y, Shang X, Zhou Y, Zhang H, Zuo L, Mei G, Xiong C, Wu T, Li H. The dose-effect relationships of cigarette and alcohol consumption with depressive symptoms: a multiple-center, cross-sectional study in 5965 Chinese middle-aged and elderly men. BMC Psychiatry. 2022 Oct 25;22(1):657. doi: 10.1186/s12888-022-04316-0.
51. Kohl IS, Luft VC, Patrão AL, Molina MDCB, Nunes MAA, Schmidt MI. Association between meatless diet and depressive episodes: A cross-sectional analysis of baseline data from the longitudinal study of adult health (ELSA-Brasil). J Affect Disord. 2023 Jan 1;320:48-56. doi: 10.1016/j.jad.2022.09.059.
52. Ortiz A, Sanches M, Abdelhack M, Schwaiger TR, Wainberg M, Tripathy SJ, Felsky D, Mulsant BH, Fiedorowicz JG. Sex-specific associations between lifetime diagnosis of bipolar disorder and cardiovascular disease: A cross-sectional analysis of 257,673 participants from the UK Biobank. J Affect Disord. 2022 Dec 15;319:663-669. doi: 10.1016/j.jad.2022.09.048.
53. Polak M, Nowicki GJ, Naylor K, Piekarski R, Ślusarska B. The Prevalence of Depression Symptoms and Their Socioeconomic and Health Predictors in a Local Community with a High Deprivation: A Cross-Sectional Studies. Int J Environ Res Public Health. 2022 Sep 19;19(18):11797. doi: 10.3390/ijerph191811797.
54. Cambron C. E-Cigarette Use Is Associated with Increased Psychological Distress among Youth: A Pooled Cross-Sectional Analysis of State-Level Data from 2019 and 2021. Int J Environ Res Public Health. 2022 Sep 17;19(18):11726. doi: 10.3390/ijerph191811726.
55. Kleppang AL, Skille EÅ. Leisure-Time Activities in Different Contexts and Depressive Symptoms in Norwegian Adolescents: A Cross-Sectional Study. Int J Environ Res Public Health. 2022 Aug 29;19(17):10769. doi: 10.3390/ijerph191710769.
56. JANAC0922: The Intersectionality of HIV-Related Stigma and Tobacco Smoking Stigma With Depressive and Anxiety Symptoms Among Women Living With HIV in the United States: A Cross-sectional Study. J Assoc Nurses AIDS Care. 2022 Sep-Oct 01;33(5):e35-e36. doi: 10.1097/JNC.0000000000000364.
57. Ki M, Shim HY, Lim J, Hwang M, Kang J, Na KS. Preventive health behaviors among people with suicide ideation using nationwide cross-sectional data in South Korea. Sci Rep. 2022 Jul 8;12(1):11615. doi: 10.1038/s41598-022-14349-w. Erratum in: Sci Rep. 2022 Aug 24;12(1):14420. doi: 10.1038/s41598-022-17355-0.
58. Iheanacho T, Maciejewski KR, Ogudebe F, Chumo F, Slade T, Leff R, Ngaruiya C. Prevalence and correlates of depression and substance use disorders in emergency department populations: A cross-sectional study at East Africa's largest public hospital. Afr J Emerg Med. 2022 Dec;12(4):307-314. doi: 10.1016/j.afjem.2022.06.008.
59. Liu S, Jiang H, Zhang D, Luo J, Zhang H. The Association between Smoking Cessation and Depressive Symptoms: Diet Quality Plays a Mediating Role. Nutrients. 2022 Jul 25;14(15):3047. doi: 10.3390/nu14153047.
60. Yadav B, Kc A, Bhusal S, Pradhan PMS. Prevalence and factors associated with symptoms of depression, anxiety and stress among traffic police officers in Kathmandu, Nepal: a cross-sectional survey. BMJ Open. 2022 Jun 7;12(6):e061534. doi: 10.1136/bmjopen-2022-061534.
61. Spooner C, Afrazi S, de Oliveira Costa J, Harris MF. Demographic and health profiles of people with severe mental illness in general practice in Australia: a cross-sectional study. Aust J Prim Health. 2022 Oct;28(5):408-416. doi: 10.1071/PY21240.
62. Han B, Volkow ND, Blanco C, Tipperman D, Einstein EB, Compton WM. Trends in Prevalence of Cigarette Smoking Among US Adults With Major Depression or Substance Use Disorders, 2006-2019. JAMA. 2022 Apr 26;327(16):1566-1576. doi: 10.1001/jama.2022.4790.
63. Nacar G, Timur Taşhan S. Eating attitudes, depressive symptoms, physical activity levels and menopausal symptoms of postmenopausal women diagnosed with coronavirus disease 2019 (COVID-19): a case-control study. Women Health. 2022 Mar;62(3):223-233. doi: 10.1080/03630242.2022.2047139.
64. Lee J, Go TH, Min S, Koh SB, Choi JR. Association between lifestyle factors and metabolic syndrome in general populations with depressive symptoms in cross-setional based cohort study of Ansung-Ansan. PLoS One. 2022 Mar 15;17(3):e0262526. doi: 10.1371/journal.pone.0262526.
65. Masaki K, Taketa RM, Nakama MK, Kawamoto CT, Pokhrel P. Relationships Between Depressive Symptoms, Anxiety, Impulsivity and Cigarette and E-cigarette Use Among Young Adults. Hawaii J Health Soc Welf. 2022 Mar;81(3):51-57.
66. Yang Q, Yang F, Zhang K. Influence of Psychological Factors on College Students' Smoking Behavior: Moderating Role of Tobacco Advertising Receptivity and Health Behavior. Am J Health Behav. 2022 Jan 31;46(1):12-28. doi: 10.5993/AJHB.46.1.2.
67. Maatouk C, Aad AM, Lucero-Prisno DE. Smoking, health risks, coping mechanisms and depression in the age of COVID-19: A cross-sectional study of the Lebanese population. J Affect Disord Rep. 2022 Apr;8:100323. doi: 10.1016/j.jadr.2022.100323.
68. Lelisho ME, Tareke SA. Prevalence and Associated Factors of Depressive Symptoms Among Mizan-Tepi University Students During the COVID-19 Pandemic. J Racial Ethn Health Disparities. 2023 Apr;10(2):633-643. doi: 10.1007/s40615-022-01251-4.
69. Pengpid S, Peltzer K. Prevalence and correlates of major depressive disorder among a national sample of middle-aged and older adults in India. Aging Ment Health. 2023 Jan;27(1):81-86. doi: 10.1080/13607863.2021.2024796.
70. Tareke SA, Lelisho ME, Hassen SS, Seid AA, Jemal SS, Teshale BM, Wotale TW, Pandey BK. The Prevalence and Predictors of Depressive, Anxiety, and Stress Symptoms Among Tepi Town Residents During the COVID-19 Pandemic Lockdown in Ethiopia. J Racial Ethn Health Disparities. 2023 Feb;10(1):43-55. doi: 10.1007/s40615-021-01195-1.
71. Kim SS, DeMarco RF. The Intersectionality of HIV-Related Stigma and Tobacco Smoking Stigma With Depressive and Anxiety Symptoms Among Women Living With HIV in the United States: A Cross-sectional Study. J Assoc Nurses AIDS Care. 2022 Sep-Oct 01;33(5):523-533.
72. Werneck AO, Peralta M, Tesler R, Marques A. Cross-sectional and prospective associations of lifestyle risk behaviors clustering with elevated depressive symptoms among middle-aged and older adults. Maturitas. 2022 Jan;155:8-13. doi: 10.1016/j.maturitas.2021.09.010.
73. Li Y, Zhang C, Ding S, Li J, Li L, Kang Y, Dong X, Wan Z, Luo Y, Cheng AS, Xie J, Duan Y. Physical activity, smoking, alcohol consumption and depressive symptoms among young, early mature and late mature people: A cross-sectional study of 76,223 in China. J Affect Disord. 2022 Feb 15;299:60-66. doi: 10.1016/j.jad.2021.11.054.
74. Grapiglia CZ, Costa JSDD, Pattussi MP, Paniz VMV, Olinto MTA. Factors associated with common mental disorders: a study based on clusters of women. Rev Saude Publica. 2021 Nov 8;55:77. doi: 10.11606/s1518-8787.2021055003124.
75. Kertes J, Neumark Y, Grunhaus L, Stein-Reisner O. Comparison of Perceptions and Smoking Cessation Experiences Between Smokers With and Without Serious Mental Illness in a Large Health Maintenance Organization. J Dual Diagn. 2021 Oct-Dec;17(4):284-295. doi: 10.1080/15504263.2021.1979348.
76. Engh JA, Ueland T, Agartz I, Andreou D, Aukrust P, Boye B, Bøen E, Drange OK, Elvsåshagen T, Hope S, Høegh MC, Joa I, Johnsen E, Kroken RA, Lagerberg TV, Lekva T, Malt UF, Melle I, Morken G, Nærland T, Steen VM, Wedervang-Resell K, Weibell MA, Westlye LT, Djurovic S, Steen NE, Andreassen OA. Plasma Levels of the Cytokines B Cell-Activating Factor (BAFF) and A Proliferation-Inducing Ligand (APRIL) in Schizophrenia, Bipolar, and Major Depressive Disorder: A Cross Sectional, Multisite Study. Schizophr Bull. 2022 Jan 21;48(1):37-46. doi: 10.1093/schbul/sbab106.
77. Alzahrani HA, Alghamdi MS, Alzahrani AA, Alzahrani AA. Tobacco smoking and depressive symptoms among male medical students in Al-Baha University. J Family Med Prim Care. 2021 Jun;10(6):2235-2240. doi: 10.4103/jfmpc.jfmpc_2315_20.
78. Cwalina SN, Pacek LR, Barrington-Trimis JL, Tackett AP, Pentz MA. Cross-Sectional Associations of Multiple Tobacco Product Use with Depressive and Anxiety Symptoms among Young Adult E-Cigarette Users. Subst Use Misuse. 2021;56(12):1807-1814. doi: 10.1080/10826084.2021.1954026.
79. Choi JS, Kwak SH, Son NH, Oh JW, Lee S, Lee EH. Sex differences in risk factors for depressive symptoms in patients with COPD: The 2014 and 2016 Korea National Health and Nutrition Examination Survey. BMC Pulm Med. 2021 May 28;21(1):180. doi: 10.1186/s12890-021-01547-x.
80. Campuzano-Cortina C, Feijoó-Fonnegra LM, Manzur-Pineda K, Palacio-Muñoz M, Rendón-Fonnegra J, Montoya L, Berrouet MC, Restrepo D. Comorbidity between depressive symptoms and substance use in-patients hospitalized for non-psychiatric diseases. Rev Colomb Psiquiatr (Engl Ed). 2021 Apr-Jun;50(2):130-137. English, Spanish. doi: 10.1016/j.rcpeng.2021.05.001.
81. Donato F, Triassi M, Loperto I, Maccaro A, Mentasti S, Crivillaro F, Elvetico A, Croce E, Raffetti E. Symptoms of mental health problems among Italian adolescents in 2017-2018 school year: a multicenter cross-sectional study. Environ Health Prev Med. 2021 Jun 21;26(1):67. doi: 10.1186/s12199-021-00988-4.
82. Bivanco-Lima D, de Souza Santos I, Wang YP, Viana MC, Andrade LH, Lotufo PA, Benseñor IJM. Cardiovascular risk factors and major depressive disorder: a cross-sectional study in São Paulo, Brazil. Sao Paulo Med J. 2021 Jul-Aug;139(4):364-371. doi: 10.1590/1516-3180.2020.0054.R1.1802021.
83. Tindimwebwa LK, Ajayi AI, Adeniyi OV. Prevalence and determinants of tobacco use amongst South African adults with mental illness in the Eastern Cape. S Afr J Psychiatr. 2021 Jun 14;27:1637. doi: 10.4102/sajpsychiatry.v27i0.1637.
84. Kleppang AL, Haugland SH, Bakken A, Stea TH. Lifestyle habits and depressive symptoms in Norwegian adolescents: a national cross-sectional study. BMC Public Health. 2021 Apr 28;21(1):816. doi: 10.1186/s12889-021-10846-1.
85. Islam MS, Rahman ME, Moonajilin MS, van Os J. Prevalence of depression, anxiety and associated factors among school going adolescents in Bangladesh: Findings from a cross-sectional study. PLoS One. 2021 Apr 1;16(4):e0247898. doi: 10.1371/journal.pone.0247898.
86. Nomura K, Minamizono S, Maeda E, Kim R, Iwata T, Hirayama J, Ono K, Fushimi M, Goto T, Mishima K, Yamamoto F. Cross-sectional survey of depressive symptoms and suicide-related ideation at a Japanese national university during the COVID-19 stay-home order. Environ Health Prev Med. 2021 Mar 5;26(1):30. doi: 10.1186/s12199-021-00953-1.
87. Qi B, Ramamurthy J, Bennani I, Trakadis YJ. Machine learning and bioinformatic analysis of brain and blood mRNA profiles in major depressive disorder: A case-control study. Am J Med Genet B Neuropsychiatr Genet. 2021 Mar;186(2):101-112. doi: 10.1002/ajmg.b.32839.
88. Piirtola M, Kaprio J, Baker TB, Piasecki TM, Piper ME, Korhonen T. The associations of smoking dependence motives with depression among daily smokers. Addiction. 2021 Aug;116(8):2162-2174. doi: 10.1111/add.15390.
89. Tariku M, Ali T, Misgana T, Hajure M, Asfaw H. Depression among Patients with Schizophrenia in Ethiopian Mental Health Hospital: Association with Sociodemographic and Clinical Variables: A Cross-Sectional Study. Depress Res Treat. 2021 Feb 9;2021:6697339. doi: 10.1155/2021/6697339.
90. Seet V, Abdin E, Asharani PV, Lee YY, Roystonn K, Wang P, Devi F, Cetty L, Teh WL, Verma S, Mok YM, Subramaniam M. Physical activity, sedentary behaviour and smoking status among psychiatric patients in Singapore - a cross-sectional study. BMC Psychiatry. 2021 Feb 18;21(1):110. doi: 10.1186/s12888-021-03103-7.
91. Peng S, Lai X, Du Y, Li Y, Tian K, Gan Y. Prevalence and Associated Factors for Depressive Symptomatology in Chinese Adults During COVID-19 Epidemic. Front Psychol. 2020 Dec 23;11:616723. doi: 10.3389/fpsyg.2020.616723.
92. Michas G, Magriplis E, Micha R, Chourdakis M, Chrousos GP, Roma E, Dimitriadis G, Panagiotakos D, Zampelas A. Sociodemographic and lifestyle determinants of depressive symptoms in a nationally representative sample of Greek adults: The Hellenic National Nutrition and Health Survey (HNNHS). J Affect Disord. 2021 Feb 15;281:192-198. doi: 10.1016/j.jad.2020.12.013.
93. Vestergaard JH, Sivapalan P, Sørensen R, Eklöf J, Achir Alispahic I, von Bülow A, Seersholm N, Jensen JS. Depressive symptoms among patients with COPD according to smoking status: a Danish nationwide case-control study of 21 184 patients. ERJ Open Res. 2020 Nov 30;6(4):00036-2020. doi: 10.1183/23120541.00036-2020.
94. Hou F, Liu H, Peng X, You L, Zhou Z, Xie H, Liu T. Gender disparities in depressive and anxiety symptoms among internal migrant workers in Shenzhen: a cross-sectional study. BMJ Open. 2020 Dec 2;10(12):e041446. doi: 10.1136/bmjopen-2020-041446.
95. Tran QA, Le VTH, Nguyen THD. Depressive symptoms and suicidal ideation among Vietnamese students aged 13-17: Results from a cross-sectional study throughout four geographical regions of Vietnam. Health Psychol Open. 2020 Nov 13;7(2):2055102920973253. doi: 10.1177/2055102920973253.
96. Fe'li SN, Yassini Ardekani SM, Dehghani A. Relationship between Serum Homocysteine and Metabolic Syndrome among Patients with Schizophrenia and Bipolar Disorder: A Cross Sectional Study. Iran J Psychiatry. 2020 Oct;15(4):266-273. doi: 10.18502/ijps.v15i4.4292.
97. Fischer CM, Hamilton AS, Slaughter RI, Milam J. A cross-sectional examination of caregiver mental health and childhood cancer survivors' tobacco, alcohol, and marijuana use. Support Care Cancer. 2021 Jul;29(7):3649-3656. doi: 10.1007/s00520-020-05861-8.
98. Sarris J, Thomson R, Hargraves F, Eaton M, de Manincor M, Veronese N, Solmi M, Stubbs B, Yung AR, Firth J. Multiple lifestyle factors and depressed mood: a cross-sectional and longitudinal analysis of the UK Biobank (N = 84,860). BMC Med. 2020 Nov 12;18(1):354. doi: 10.1186/s12916-020-01813-5.
99. Bahta M, Berhe T, Russom M, Tesfamariam EH, Ogbaghebriel A. Magnitude, Nature, and Risk Factors of Adverse Drug Reactions Associated with First Generation Antipsychotics in Outpatients with Schizophrenia: A Cross-Sectional Study. Integr Pharm Res Pract. 2020 Oct 14;9:205-217. doi: 10.2147/IPRP.S271814.
100. Lee S, Oh Y, Kim H, Kong M, Moon J. Implications of electronic cigarette use for depressive mood: A nationwide cross-sectional study. Medicine (Baltimore). 2020 Oct 2;99(40):e22514. doi: 10.1097/MD.0000000000022514.
101. Uju Y, Kanzaki T, Yamasaki Y, Kondo T, Nanasawa H, Takeuchi Y, Yanagisawa Y, Kusanishi S, Nakano C, Enomoto T, Sako A, Yanai H, Mishima S, Mimori S, Igarashi K, Takizawa T, Hayakawa T. A cross-sectional study on metabolic similarities and differences between inpatients with schizophrenia and those with mood disorders. Ann Gen Psychiatry. 2020 Sep 22;19:53. doi: 10.1186/s12991-020-00303-5.
102. Astutik E, Hidajah AC, Tama TD, Efendi F, Li CY. Prevalence and determinants of depressive symptoms among adults in Indonesia: A cross-sectional population-based national survey. Nurs Forum. 2021 Jan;56(1):37-44. doi: 10.1111/nuf.12508.
103. Sunderland M, Champion K, Slade T, Chapman C, Newton N, Thornton L, Kay-Lambkin F, McBride N, Allsop S, Parmenter B, Teesson M; Health4Life Team. Age-varying associations between lifestyle risk factors and major depressive disorder: a nationally representative cross-sectional study of adolescents. Soc Psychiatry Psychiatr Epidemiol. 2021 Jan;56(1):129-139. doi: 10.1007/s00127-020-01888-8.
104. Jang BN, Lee HJ, Joo JH, Park EC, Jang SI. Association between health behaviours and depression: findings from a national cross-sectional study in South Korea. BMC Psychiatry. 2020 May 14;20(1):238. doi: 10.1186/s12888-020-02628-7.
105. Khobragade B, Sharma V, Deshpande SN. A study on tobacco use in women with major mental illnesses- schizophrenia, bipolar disorder and recurrent depression. Psychiatry Res. 2020 Aug;290:113121. doi: 10.1016/j.psychres.2020.113121.
106. Wei S, Wang D, Wei G, Wang J, Zhou H, Xu H, Xia L, Tian Y, Dai Q, Zhu R, Wang W, Chen D, Xiu M, Wang L, Zhang XY. Association of cigarette smoking with cognitive impairment in male patients with chronic schizophrenia. Psychopharmacology (Berl). 2020 Nov;237(11):3409-3416. doi: 10.1007/s00213-020-05621-w.
107. Desalegn D, Girma S, Tessema W, Yeshigeta E, Kebeta T. Quality of Life and Associated Factors among Patients with Schizophrenia Attending Follow-Up Treatment at Jimma Medical Center, Southwest Ethiopia: A Cross-Sectional Study. Psychiatry J. 2020 Aug 21;2020:4065082. doi: 10.1155/2020/4065082.
108. Robles B, Jewell MP, Thomas Tobin CS, Smith LV, Kuo T. Varying levels of depressive symptoms and lifestyle health behaviors in a low income, urban population. J Behav Med. 2021 Apr;44(2):212-221. doi: 10.1007/s10865-020-00179-6.
109. Ren Z, Wang Q, Xiong W, Fan X, Guo X, Ma J, He M, Shen Y, Zhou G, Gong P, Liu M, Yang X, Liu H, Zhang X. Association between lifestyle factors and depressive symptoms among Chinese middle school students: a cross-sectional study. Psychol Health Med. 2021 Dec;26(10):1266-1273. doi: 10.1080/13548506.2020.1823441.
110. Bae SM. The association between health-related factors, physical and mental diseases, social activities, and cognitive function in elderly Koreans: a population-based cross-sectional study. Psychogeriatrics. 2020 Sep;20(5):654-662. doi: 10.1111/psyg.12561.
111. Desalegn D, Girma S, Abdeta T. Quality of life and its association with current substance use, medication non-adherence and clinical factors of people with schizophrenia in Southwest Ethiopia: a hospital-based cross-sectional study. Health Qual Life Outcomes. 2020 Mar 30;18(1):82. doi: 10.1186/s12955-020-01340-0.
112. Kelly ME, Guillot CR, Quinn EN, Lucke HR, Bello MS, Pang RD, Leventhal AM. Anxiety sensitivity in relation to cigarette smoking and other substance use in African American smokers. Psychol Addict Behav. 2020 Sep;34(6):669-679. doi: 10.1037/adb0000573.
113. Jacob L, Smith L, Jackson SE, Haro JM, Shin JI, Koyanagi A. Secondhand Smoking and Depressive Symptoms Among In-School Adolescents. Am J Prev Med. 2020 May;58(5):613-621. doi: 10.1016/j.amepre.2019.12.008.
114. Mayro EL, Murchison AP, Hark LA, Silverstein M, Wang OY, Gilligan JP, Leiby BE, Pizzi LT, Casten RJ, Rovner BW, Haller JA. Prevalence of depressive symptoms and associated factors in an urban, ophthalmic population. Eur J Ophthalmol. 2021 Mar;31(2):740-747. doi: 10.1177/1120672120901701.
115. Mo PK, Li JB, Jiang H, Lau JTF. Problematic Internet Use and Smoking among Chinese Junior Secondary Students: The Mediating Role of Depressive Symptomatology and Family Support. Int J Environ Res Public Health. 2019 Dec 11;16(24):5053. doi: 10.3390/ijerph16245053.
116. Noguchi T, Nakagawa-Senda H, Tamai Y, Nishiyama T, Watanabe M, Hosono A, Shibata K, Ichikawa M, Wakabayashi R, Nagaya K, Ema K, Okamoto N, Tsujimura S, Fujita H, Kamiya M, Kondo F, Yamada T, Suzuki S. Association Between Second-hand Smoke Exposure and Depressive Symptoms Among Japanese Adults: A Cross-sectional Study. J Epidemiol. 2020 Dec 5;30(12):566-573. doi: 10.2188/jea.JE20190146.
117. Charoensakulchai S, Usawachoke S, Kongbangpor W, Thanavirun P, Mitsiriswat A, Pinijnai O, Kaensingh S, Chaiyakham N, Chamnanmont C, Ninnakala N, Hiri-O-Tappa P, Ponginsee V, Atichatpongsuk V, Asawathepmetha EO, Thongprayoon C, Mao MA, Cheungpasitporn W, Varothai N, Kaewput W. Prevalence and associated factors influencing depression in older adults living in rural Thailand: A cross-sectional study. Geriatr Gerontol Int. 2019 Dec;19(12):1248-1253. doi: 10.1111/ggi.13804.
118. Fang Y, Wang W, Zhu C, Lin GN, Cheng Y, Zou J, Cui D. Use of tobacco in schizophrenia: A double-edged sword. Brain Behav. 2019 Nov;9(11):e01433. doi: 10.1002/brb3.1433.
119. Rasouli N, Malakouti SK, Rezaeian M, Saberi SM, Nojomi M, De Leo D, Ramezani-Farani A. Risk Factors of Suicide Death Based on Psychological Autopsy Method; a Case-Control Study. Arch Acad Emerg Med. 2019 Sep 1;7(1):e50.
120. Pham T, Williams JVA, Bhattarai A, Dores AK, Isherwood LJ, Patten SB. Electronic cigarette use and mental health: A Canadian population-based study. J Affect Disord. 2020 Jan 1;260:646-652. doi: 10.1016/j.jad.2019.09.026.
121. Petroulia I, Kyriakos CN, Papadakis S, Tzavara C, Filippidis FT, Girvalaki C, Peleki T, Katsaounou P, McNeill A, Mons U, Fernández E, Demjén T, Trofor AC, Herbeć A, Zatoński WA, Tountas Y, Fong GT, Vardavas CI; EUREST-PLUS consortium. Patterns of tobacco use, quit attempts, readiness to quit and self-efficacy among smokers with anxiety or depression: Findings among six countries of the EUREST-PLUS ITC Europe Surveys. Tob Induc Dis. 2019 Feb 5;16:A9. doi: 10.18332/tid/98965. Erratum in: Tob Induc Dis. 2019 Nov 22;17:83. doi: 10.18332/tid/114415.
122. Morlet E, Costemale-Lacoste JF, Poulet E, McMahon K, Hoertel N, Limosin F; CSA Study Group. Psychiatric and physical outcomes of long-term use of lithium in older adults with bipolar disorder and major depressive disorder: A cross-sectional multicenter study. J Affect Disord. 2019 Dec 1;259:210-217. doi: 10.1016/j.jad.2019.08.056.
123. Yazew KG, Beshah DT, Salih MH, Zeleke TA. Factors Associated with Depression among Heart Failure Patients at Cardiac Follow-Up Clinics in Northwest Ethiopia, 2017: A Cross-Sectional Study. Psychiatry J. 2019 Jul 21;2019:6892623.
124. Hestetun I, Svendsen MV, Oellingrath IM. Lifestyle, appearance satisfaction and depressive symptoms in 13-16 years old Norwegian adolescents - A cross-sectional study. Nord J Psychiatry. 2019 Nov;73(8):482-489. doi: 10.1080/08039488.2019.1653964.
125. Werneck AO, Oyeyemi AL, Szwarcwald CL, Stubbs B, Silva DR. Potential influence of physical, psychological and lifestyle factors on the association between television viewing and depressive symptoms: A cross-sectional study. Gen Hosp Psychiatry. 2019 Sep-Oct;60:37-43. doi: 10.1016/j.genhosppsych.2019.07.005.
126. Kozak K, Lowe DJE, George TP. Effects of Tobacco Smoking Status on Verbal Learning and Memory in Patients With Schizophrenia and Non-Psychiatric Controls. Am J Addict. 2019 Nov;28(6):503-511. doi: 10.1111/ajad.12903.
127. Azzam P, Obeid S, Haddad C, Kazour F, Nabbout R, Hallit S, Azar J. Relationship Between Impulsivity and Clinical and Sociodemographic Variables Among Lebanese Patients With Bipolar Disorder: Results of a Cross-Sectional Study. J Nerv Ment Dis. 2019 Jul;207(7):596-603. doi: 10.1097/NMD.0000000000001012.
128. Siqueira-Campos VME, Da Luz RA, de Deus JM, Martinez EZ, Conde DM. Anxiety and depression in women with and without chronic pelvic pain: prevalence and associated factors. J Pain Res. 2019 Apr 16;12:1223-1233. doi: 10.2147/JPR.S195317.
129. Abd Rashid R, Kanagasundram S, Danaee M, Abdul Majid H, Sulaiman AH, Ahmad Zahari MM, Ng CG, Francis B, Wan Husin WAI, Su TT. The Prevalence of Smoking, Determinants and Chance of Psychological Problems among Smokers in an Urban Community Housing Project in Malaysia. Int J Environ Res Public Health. 2019 May 18;16(10):1762. doi: 10.3390/ijerph16101762.
130. Tran BX, Vu GT, Pham KTH, Vuong QH, Ho MT, Vuong TT, Nguyen HT, Nguyen CT, Latkin CA, Ho CSH, Ho RCM. Depressive Symptoms among Industrial Workers in Vietnam and Correlated Factors: A Multi-Site Survey. Int J Environ Res Public Health. 2019 May 11;16(9):1642. doi: 10.3390/ijerph16091642.
131. Guillot CR, Blackledge SM, Douglas ME, Cloutier RM, Liautaud MM, Pang RD, Kirkpatrick MG, Leventhal AM. Indirect Associations of Anxiety Sensitivity with Tobacco, Alcohol, and Other Drug Use Problems Through Emotional Disorder Symptoms in Adolescents. Behav Med. 2020 Apr-Jun;46(2):161-169. doi: 10.1080/08964289.2019.1573797.
132. Harris T, Winetrobe H, Rhoades H, Wenzel S. The Role of Mental Health and Substance Use in Homeless Adults' Tobacco Use and Cessation Attempts. J Dual Diagn. 2019 Apr-Jun;15(2):76-87. doi: 10.1080/15504263.2019.1579947.
133. Khorasanchi Z, Bahrami A, Avan A, Jaberi N, Rezaey M, Bahrami-Taghanaki H, Ferns GA, Ghayour-Mobarhan M. Passive smoking is associated with cognitive and emotional impairment in adolescent girls. J Gen Psychol. 2019 Jan-Mar;146(1):68-78. doi: 10.1080/00221309.2018.1535485.
134. Alharbi H, Almalki A, Alabdan F, Haddad B. Depression among medical students in Saudi medical colleges: a cross-sectional study. Adv Med Educ Pract. 2018 Dec 4;9:887-891. doi: 10.2147/AMEP.S182960.
135. Stramecki F, Kotowicz KD, Piotrowski P, Frydecka D, Rymaszewska J, Beszłej JA, Samochowiec J, Jabłoński M, Wroński M, Moustafa AA, Misiak B. Assessment of the Association Between Cigarette Smoking and Cognitive Performance in Patients With Schizophrenia-Spectrum Disorders: A Case-Control Study. Front Psychiatry. 2018 Dec 3;9:642. doi: 10.3389/fpsyt.2018.00642.
136. Elamoshy R, Bird Y, Thorpe LU, Moraros J. Examining the association between diabetes, depressive symptoms, and suicidal ideation among Aboriginal Canadian peoples living off-reserve: a cross-sectional, population-based study. Diabetes Metab Syndr Obes. 2018 Nov 22;11:767-780. doi: 10.2147/DMSO.S184058.
137. Lee J, Kim TH, Min S, Kim MH, Park KC, Moon JS, Ahn JS. Depressive symptoms and suicidal behaviours in adolescent non-daily smokers compared to daily smokers and never-smokers in Korea: National cross-sectional study. PLoS One. 2018 Nov 14;13(11):e0207182. doi: 10.1371/journal.pone.0207182.
138. Smith PH, Chhipa M, Bystrik J, Roy J, Goodwin RD, McKee SA. Cigarette smoking among those with mental disorders in the US population: 2012-2013 update. Tob Control. 2020 Jan;29(1):29-35. doi: 10.1136/tobaccocontrol-2018-054268.
139. Wiernik E, Airagnes G, Lequy E, Gomajee R, Melchior M, Le Faou AL, Limosin F, Goldberg M, Zins M, Lemogne C. Electronic cigarette use is associated with depressive symptoms among smokers and former smokers: Cross-sectional and longitudinal findings from the Constances cohort. Addict Behav. 2019 Mar;90:85-91. doi: 10.1016/j.addbeh.2018.10.021.
140. Ward HB, Lawson MT, Addington J, Bearden CE, Cadenhead KS, Cannon TD, Cornblatt BA, Jeffries CD, Mathalon DH, McGlashan TH, Seidman LJ, Tsuang MT, Walker EF, Woods SW, Perkins DO. Tobacco use and psychosis risk in persons at clinical high risk. Early Interv Psychiatry. 2019 Oct;13(5):1173-1181. doi: 10.1111/eip.12751.
141. Abu Nazel M, Elkady H, Abd Allah S, Ibrahim R. Pattern and correlates of tobacco smoking among mentally ill male patients in El-Mamoura Psychiatric Hospital, Alexandria, Egypt. J Egypt Public Health Assoc. 2017 Sep 1;92(3):167-176.
142. Mallet J, Mazer N, Dubertret C, Le Strat Y. Tobacco Smoking and Psychotic-Like Experiences in a General Population Sample. J Clin Psychiatry. 2018 Oct 2;79(6):17m11994. doi: 10.4088/JCP.17m11994.
143. Asai Y, Obayashi K, Oume M, Ogura M, Takeuchi K, Yamagami Y, Tai Y, Kurumatani N, Saeki K. Farming habit, light exposure, physical activity, and depressive symptoms. A cross-sectional study of the HEIJO-KYO cohort. J Affect Disord. 2018 Dec 1;241:235-240. doi: 10.1016/j.jad.2018.08.003.
144. Klakk H, Kristensen PL, Andersen LB, Froberg K, Møller NC, Grøntved A. Symptoms of depression in young adulthood is associated with unfavorable clinical- and behavioral cardiovascular disease risk factors. Prev Med Rep. 2018 May 22;11:209-215. doi: 10.1016/j.pmedr.2018.05.017.
145. Aguocha C, Uwakwe R, Olose E, Amadi K, Onyeama G, Duru C. Clinical implication of smoking among patients with schizophrenia at a Tertiary Institution in South East Nigeria. Afr Health Sci. 2018 Mar;18(1):102-110. doi: 10.4314/ahs.v18i1.14.
146. Chee GL, Wynaden D, Heslop K. The provision of physical health care by nurses to young people with first episode psychosis: A cross-sectional study. J Psychiatr Ment Health Nurs. 2018 Sep;25(7):411-422. doi: 10.1111/jpm.12487.
147. Stubbs B, Vancampfort D, Firth J, Solmi M, Siddiqi N, Smith L, Carvalho AF, Koyanagi A. Association between depression and smoking: A global perspective from 48 low- and middle-income countries. J Psychiatr Res. 2018 Aug;103:142-149. doi: 10.1016/j.jpsychires.2018.05.018.
148. Stubbs B, Vancampfort D, Firth J, Solmi M, Siddiqi N, Smith L, Carvalho AF, Koyanagi A. Association between depression and smoking: A global perspective from 48 low- and middle-income countries. J Psychiatr Res. 2018 Aug;103:142-149. doi: 10.1016/j.jpsychires.2018.05.018.
149. Alosaimi FD, Abalhassan M, Alhaddad B, Fallata EO, Alhabbad A, Alshenqiti R, Alassiry MZ. Gender differences and risk factors for smoking among patients with various psychiatric disorders in Saudi Arabia: a cross-sectional study. Int J Ment Health Syst. 2018 May 3;12:21. doi: 10.1186/s13033-018-0201-7.
150. Furihata R, Konno C, Suzuki M, Takahashi S, Kaneita Y, Ohida T, Uchiyama M. Unhealthy lifestyle factors and depressive symptoms: A Japanese general adult population survey. J Affect Disord. 2018 Jul;234:156-161. doi: 10.1016/j.jad.2018.02.093.
151. Liu Q, Cai H, Yang LH, Xiang YB, Yang G, Li H, Gao YT, Zheng W, Susser E, Shu XO. Depressive symptoms and their association with social determinants and chronic diseases in middle-aged and elderly Chinese people. Sci Rep. 2018 Mar 1;8(1):3841. doi: 10.1038/s41598-018-22175-2.
152. Rodriquez EJ, Livaudais-Toman J, Gregorich SE, Jackson JS, Nápoles AM, Pérez-Stable EJ. Relationships between allostatic load, unhealthy behaviors, and depressive disorder in U.S. adults, 2005-2012 NHANES. Prev Med. 2018 May;110:9-15. doi: 10.1016/j.ypmed.2018.02.002.
153. Mallet J, Le Strat Y, Schürhoff F, Mazer N, Portalier C, Andrianarisoa M, Aouizerate B, Berna F, Brunel L, Capdevielle D, Chereau I, D'Amato T, Dubreucq J, Faget C, Gabayet F, Honciuc RM, Lançon C, Llorca PM, Misdrahi D, Rey R, Roux P, Schandrin A, Urbach M, Vidailhet P, Fond G, Dubertret C; FACE-SZ (FondaMental Academic Centers of Expertise for Schizophrenia) group. Tobacco smoking is associated with antipsychotic medication, physical aggressiveness, and alcohol use disorder in schizophrenia: results from the FACE-SZ national cohort. Eur Arch Psychiatry Clin Neurosci. 2019 Jun;269(4):449-457. doi: 10.1007/s00406-018-0873-7.
154. Medeiros GC, Lafer B, Kapczinski F, Miranda-Scippa Â, Almeida KM. Bipolar disorder and tobacco smoking: Categorical and dimensional clinical correlates in subjects from the Brazilian bipolar research network. Compr Psychiatry. 2018 Apr;82:14-21. doi: 10.1016/j.comppsych.2017.12.003.
155. Jaalouk D, Matar Boumosleh J, Helou L, Abou Jaoude M. Dietary patterns, their covariates, and associations with severity of depressive symptoms among university students in Lebanon: a cross-sectional study. Eur J Nutr. 2019 Apr;58(3):997-1008. doi: 10.1007/s00394-018-1614-4.
156. Athar H, Mukhtar N, Shah S, Mukhtar F. Depression And Associated Factors: A Cross-Sectional Study Using Beck Depression Inventory. J Ayub Med Coll Abbottabad. 2017 Oct-Dec;29(4):667-670.
157. Chiu M, Rahman F, Vigod S, Wilton AS, Kurdyak P. Temporal trends in cardiovascular disease risk factor profiles in a population-based schizophrenia sample: a repeat cross-sectional study. J Epidemiol Community Health. 2018 Jan;72(1):71-77. doi: 10.1136/jech-2017-209565.
158. Veromaa V, Kautiainen H, Korhonen PE. Physical and mental health factors associated with work engagement among Finnish female municipal employees: a cross-sectional study. BMJ Open. 2017 Oct 5;7(10):e017303. doi: 10.1136/bmjopen-2017-017303.
159. Mallet J, Le Strat Y, Schürhoff F, Mazer N, Portalier C, Andrianarisoa M, Aouizerate B, Berna F, Brunel L, Capdevielle D, Chereau I, D'Amato T, Denizot H, Dubreucq J, Faget C, Gabayet F, Lançon C, Llorca PM, Misdrahi D, Rey R, Roux P, Schandrin A, Urbach M, Vidailhet P, Fond G, Dubertret C; FACE-SZ (FondaMental Academic Centers of Expertise for Schizophrenia) group. Cigarette smoking and schizophrenia: a specific clinical and therapeutic profile? Results from the FACE-Schizophrenia cohort. Prog Neuropsychopharmacol Biol Psychiatry. 2017 Oct 3;79(Pt B):332-339. doi: 10.1016/j.pnpbp.2017.06.026.
160. Bhavsar V, Jauhar S, Murray RM, Hotopf M, Hatch SL, McNeill A, Boydell J, MacCabe JH. Tobacco smoking is associated with psychotic experiences in the general population of South London. Psychol Med. 2018 Jan;48(1):123-131. doi: 10.1017/S0033291717001556.

***Related to other disease***

1. Bhavsar V, Jauhar S, Murray RM, Hotopf M, Hatch SL, McNeill A, Boydell J, MacCabe JH. Tobacco smoking is associated with psychotic experiences in the general population of South London. Psychol Med. 2018 Jan;48(1):123-131. doi: 10.1017/S0033291717001556.
2. Sullivan SA, Kounali D, Lilford P, Morris R, Kessler D, Hamilton W, Lewis G, Nazareth I. External validation of a prognostic model to improve prediction of psychosis in primary care. Br J Gen Pract. 2024 Jul 15:BJGP.2024.0017. doi: 10.3399/BJGP.2024.0017.
3. Ban KF, Rogers E, Khan M, Scheidell J, Charles D, Bryant KJ, Justice AC, Braithwaite RS, Caniglia EC. Does smoking cessation reduce other substance use, psychiatric symptoms, and pain symptoms? Results from an emulated hypothetical randomized trial of US veterans. PLoS One. 2024 Jul 3;19(7):e0298576. doi: 10.1371/journal.pone.0298576.
4. Triolo F, Vetrano DL, Trevisan C, Sjöberg L, Calderón-Larrañaga A, Belvederi Murri M, Fratiglioni L, Dekhtyar S. Mapping 15-year depressive symptom transitions in late life: population-based cohort study. Br J Psychiatry. 2024 May 30:1-7. doi: 10.1192/bjp.2024.84.
5. Hines LA, Heron J, Zammit S. Incident psychotic experiences following self-reported use of high-potency cannabis: Results from a longitudinal cohort study. Addiction. 2024 Sep;119(9):1629-1634. doi: 10.1111/add.16517.
6. Gambhir T, Al Snih S. Cardiovascular Disease, Depressive Symptoms, and Heart Failure in Mexican American Aged 75 Years and Older During 12 Years of Follow Up. J Affect Disord Rep. 2024 Apr;16:100724. doi: 10.1016/j.jadr.2024.100724.
7. De Micheli A, Provenzani U, Krakowski K, Oliver D, Damiani S, Brondino N, McGuire P, Fusar-Poli P. Physical Health and Transition to Psychosis in People at Clinical High Risk. Biomedicines. 2024 Feb 26;12(3):523. doi: 10.3390/biomedicines12030523.
8. Lee PH, Tervo-Clemmens B, Liu RT, Gersten MB, Jung JY, Janes AC, Gilman J. Use of Tobacco Products and Suicide Attempts Among Elementary School-Aged Children. JAMA Netw Open. 2024 Feb 5;7(2):e240376. doi: 10.1001/jamanetworkopen.2024.0376.
9. Mittal S, Komiyama M, Ozaki Y, Yamakage H, Satoh-Asahara N, Wada H, Yasoda A, Funamoto M, Katanasaka Y, Sunagawa Y, Morimoto T, Akao M, Abe M, Takahashi Y, Nakayama T, Hasegawa K. Impact of smoking initiation age on nicotine dependency and cardiovascular risk factors: a retrospective cohort study in Japan. Eur Heart J Open. 2023 Dec 20;4(1):oead135. doi: 10.1093/ehjopen/oead135.
10. Jaarsma E, Nooyens A, Kok AAL, Köhler S, van Boxtel M, Verschuren WMM, Huisman M. Modifiable Risk Factors for Accelerated Decline in Processing Speed: Results from Three Dutch Population Cohorts. J Prev Alzheimers Dis. 2024;11(1):108-116. doi: 10.14283/jpad.2023.64.
11. Matta J, Robineau O, Wiernik E, Carrat F, Severi G, Touvier M, Gouraud C, Ouazana Vedrines C, Pitron V, Ranque B, Pignon B, Hoertel N, Kab S, Goldberg M, Zins M, Lemogne C. Depression and anxiety before and at the beginning of the COVID-19 pandemic and incident persistent symptoms: a prospective population-based cohort study. Mol Psychiatry. 2023 Oct;28(10):4261-4271. doi: 10.1038/s41380-023-02179-9.
12. Pasch KE, Thomas JE, North C, Marti CN, Loukas A. Exposure to tobacco retail outlet tobacco marketing and initiation of cigarette and e-cigarette use: Depressive symptoms as a moderator. Drug Alcohol Depend. 2023 Jul 1;248:109935. doi: 10.1016/j.drugalcdep.2023.109935.
13. Ohta T, Sasai H, Osuka Y, Kojima N, Abe T, Yamashita M, Obuchi SP, Ishizaki T, Fujiwara Y, Awata S, Toba K; IRIDE Cohort Study Investigators. Age- and sex-specific associations between sarcopenia severity and poor cognitive function among community-dwelling older adults in Japan: The IRIDE Cohort Study. Front Public Health. 2023 Apr 4;11:1148404. doi: 10.3389/fpubh.2023.1148404.
14. Rantanen AT, Kautiainen H, Korhonen PE. Depressive symptoms and mortality - effect variation by body mass index: a prospective study in a primary care population. Int J Obes (Lond). 2023 Jun;47(6):512-519. doi: 10.1038/s41366-023-01296-3.
15. Akaishi T, Tarasawa K, Fushimi K, Hamada H, Saito M, Kobayashi N, Kikuchi S, Tomita H, Ishii T, Fujimori K, Yaegashi N. Risk Factors Associated With Peripartum Suicide Attempts in Japan. JAMA Netw Open. 2023 Jan 3;6(1):e2250661. doi: 10.1001/jamanetworkopen.2022.50661.
16. Sulkava S, Haukka J, Sulkava R, Laatikainen T, Paunio T. Association Between Psychological Distress and Incident Dementia in a Population-Based Cohort in Finland. JAMA Netw Open. 2022 Dec 1;5(12):e2247115. doi: 10.1001/jamanetworkopen.2022.47115.
17. Gustad LT, Holand AM, Hynnekleiv T, Bjerkeset O, Berk M, Romundstad S. The bidirectional association between depressive symptoms, assessed by the HADS, and albuminuria-A longitudinal population-based cohort study with repeated measures from the HUNT2 and HUNT3 Study. PLoS One. 2022 Sep 15;17(9):e0274271. doi: 10.1371/journal.pone.0274271.
18. Murray AL, Taut D, Baban A, Hemady CL, Walker S, Osafo J, Sikander S, Tomlinson M, Toit SD, Marlow M, Ward CL, Fernando A, Madrid B, Van Thang V, Tuyen HD, Dunne M, Hughes C, Fearon P, Valdebenito S, Eisner M. Associations Between ADHD Symptoms and Maternal and Birth Outcomes: An Exploratory Analysis in a Multi-Country Cohort of Expectant Mothers. J Atten Disord. 2022 Dec;26(14):1882-1894. doi: 10.1177/10870547221105064.
19. Denissoff A, Mustonen A, Miettunen J, Alakokkare AE, Veijola J, Scott JG, Sami MB, Niemelä S. Trajectories of adolescent psychotic-like experiences and early cannabis exposure: Results from a Finnish Birth Cohort Study. Schizophr Res. 2022 Aug;246:95-102. doi: 10.1016/j.schres.2022.06.014.
20. Tsukahara M, So R, Yada Y, Kodama M, Kishi Y, Yamada N. Changes in Plasma Clozapine Levels after Smoking Cessation in Japanese Inpatients with Schizophrenia: A Retrospective Cohort Study. Acta Med Okayama. 2022 Apr;76(2):137-143. doi: 10.18926/AMO/63407.
21. van der Heijden HS, Schirmbeck F, Berry L, Simons CJP, Bartels-Velthuis AA, Bruggeman R, de Haan L, Vermeulen J. Impact of coping styles on substance use in persons with psychosis, siblings, and controls. Schizophr Res. 2022 Mar;241:102-109. doi: 10.1016/j.schres.2022.01.030.
22. Baranyi G, Sieber S, Pearce J, Cullati S, Dibben C, Cheval B. Lower social participation partly explains the association between perceived neighbourhood crime and depressive symptoms in European adults aged 50 years or older: A longitudinal mediation analysis. Prev Med. 2022 Feb;155:106954. doi: 10.1016/j.ypmed.2022.106954.
23. Falcaro M, Osborn D, Hayes J, Coyle G, Couperthwaite L, Weich S, Walters KR. Time trends in access to smoking cessation support for people with depression or severe mental illness: a cohort study in English primary care. BMJ Open. 2021 Dec 3;11(12):e048341. doi: 10.1136/bmjopen-2020-048341.
24. Foroughi M, Medina Inojosa JR, Lopez-Jimenez F, Saeidifard F, Suarez L, Stokin GB, Prieto ML, Rocca WA, Frye MA, Morgan RJ. Association of Bipolar Disorder With Major Adverse Cardiovascular Events: A Population-Based Historical Cohort Study. Psychosom Med. 2022 Jan 1;84(1):97-103. doi: 10.1097/PSY.0000000000001017.
25. van der Heijden HS, Schirmbeck F, Kempton MJ, van der Gaag M, Allott K, Nelson B, Ruhrmann S, de Haan L, Vermeulen JM; Eu-Gei High Risk Study. Impact of smoking Behavior on cognitive functioning in persons at risk for psychosis and healthy controls: A longitudinal study. Eur Psychiatry. 2021 Sep 21;64(1):e60. doi: 10.1192/j.eurpsy.2021.2233.
26. Werneck AO, Stubbs B, Kandola A, Hamer M, Silva DR. Prospective associations of different contexts of physical activity with psychological distress and well-being among middle-aged adults: An analysis of the 1970 British Cohort Study. J Psychiatr Res. 2021 Aug;140:15-21. doi: 10.1016/j.jpsychires.2021.05.049.
27. Brannigan R, Tanskanen A, Huttunen MO, Cannon M, Leacy FP, Clarke MC. Maternal smoking during pregnancy and offspring psychiatric disorder: a longitudinal birth cohort study. Soc Psychiatry Psychiatr Epidemiol. 2022 Mar;57(3):595-600. doi: 10.1007/s00127-021-02094-w.
28. Beunders AJM, Kok AAL, Kosmas PC, Beekman ATF, Sonnenberg CM, Schouws SNTM, Kupka RW, Stek ML, Dols A. Physical comorbidity in Older-Age Bipolar Disorder (OABD) compared to the general population - a 3-year longitudinal prospective cohort study. J Affect Disord. 2021 Jun 1;288:83-91. doi: 10.1016/j.jad.2021.03.057.
29. Chaturvedi J, Sabbah W, Gallagher JE, Turner J, Curl C, Stewart R. Hospital admissions for dental disorders in patients with severe mental illness in Southeast London: A register-based cohort study. Eur J Oral Sci. 2021 Feb;129(1):e12752. doi: 10.1111/eos.12752.
30. Li A, Rosella LC, Kurdyak P, Wodchis WP. Depression as a Risk Factor for Physical Illness and Multimorbidity in a Cohort with No Prior Comorbidity. Can J Psychiatry. 2021 Aug;66(8):726-736. doi: 10.1177/0706743720974832.
31. Yokoyama M, Tanaka K, Sugiyama T, Arakawa M, Miyake Y. Cesarean section is associated with increased risk of postpartum depressive symptoms in Japan: the Kyushu Okinawa Maternal and Child Health Study. J Affect Disord. 2021 Jan 1;278:497-501. doi: 10.1016/j.jad.2020.09.106.
32. Kubota C, Inada T, Shiino T, Ando M, Sato M, Nakamura Y, Yamauchi A, Morikawa M, Okada T, Ohara M, Aleksic B, Murase S, Goto S, Kanai A, Ozaki N. The Risk Factors Predicting Suicidal Ideation Among Perinatal Women in Japan. Front Psychiatry. 2020 May 15;11:441. doi: 10.3389/fpsyt.2020.00441.
33. Costa FO, Cortelli JR, Lima RPE, Costa AA, Cortelli SC, Cota LOM. Depressive disorders associated with the recurrence of periodontitis in periodontal maintenance. J Int Acad Periodontol. 2020 Apr 1;22(2):1-9.
34. Carney H, Marrie RA, Bolton JM, Patten SB, Graff LA, Bernstein CN, Kowalec K. Prevalence and Risk Factors of Substance Use Disorder in Inflammatory Bowel Disease. Inflamm Bowel Dis. 2021 Jan 1;27(1):58-64. doi: 10.1093/ibd/izaa014.
35. Amos R, Manalastas EJ, White R, Bos H, Patalay P. Mental health, social adversity, and health-related outcomes in sexual minority adolescents: a contemporary national cohort study. Lancet Child Adolesc Health. 2020 Jan;4(1):36-45. doi: 10.1016/S2352-4642(19)30339-6.
36. Krieger I, Tzur Bitan D, Comaneshter D, Cohen A, Feingold D. Increased risk of smoking-related illnesses in schizophrenia patients: A nationwide cohort study. Schizophr Res. 2019 Oct;212:121-125. doi: 10.1016/j.schres.2019.07.058.
37. Krieger I, Tzur Bitan D, Comaneshter D, Cohen A, Feingold D. Increased risk of smoking-related illnesses in schizophrenia patients: A nationwide cohort study. Schizophr Res. 2019 Oct;212:121-125. doi: 10.1016/j.schres.2019.07.058.
38. Tang X, Lu Z, Hu D, Zhong X. Influencing factors for prenatal Stress, anxiety and depression in early pregnancy among women in Chongqing, China. J Affect Disord. 2019 Jun 15;253:292-302. doi: 10.1016/j.jad.2019.05.003.
39. Fujita T, Babazono A, Harano Y, Jiang P. Risk of depressive disorders after tobacco smoking cessation: a retrospective cohort study in Fukuoka, Japan. BMJ Open. 2019 Mar 23;9(3):e025124. doi: 10.1136/bmjopen-2018-025124.
40. Vermeulen J, Schirmbeck F, Blankers M, van Tricht M, van den Brink W, de Haan L; Genetic Risk and Outcome of Psychosis (GROUP) investigators. Smoking, symptoms, and quality of life in patients with psychosis, siblings, and healthy controls: a prospective, longitudinal cohort study. Lancet Psychiatry. 2019 Jan;6(1):25-34. doi: 10.1016/S2215-0366(18)30424-3.
41. Song C, Li W, Leng J, Wang L, Li W, Shi F, Liu G, Zhou J, Yang X. Passive smoking and postpartum depression among Chinese women: A prospective cohort study in Tianjin, China. Women Health. 2019 Mar;59(3):281-293. doi: 10.1080/03630242.2018.1478365.
42. Kobayashi LC, Steptoe A. Social Isolation, Loneliness, and Health Behaviors at Older Ages: Longitudinal Cohort Study. Ann Behav Med. 2018 May 31;52(7):582-593. doi: 10.1093/abm/kax033.
43. Lee H, Myung W, Lee C, Choi J, Kim H, Carroll BJ, Kim DK. Clinical epidemiology of long-term suicide risk in a nationwide population-based cohort study in South Korea. J Psychiatr Res. 2018 May;100:47-55. doi: 10.1016/j.jpsychires.2018.01.018.
44. Jones HJ, Gage SH, Heron J, Hickman M, Lewis G, Munafò MR, Zammit S. Association of Combined Patterns of Tobacco and Cannabis Use in Adolescence With Psychotic Experiences. JAMA Psychiatry. 2018 Mar 1;75(3):240-246. doi: 10.1001/jamapsychiatry.2017.4271.
45. Song YM, Lee K, Sung J. Genetic and environmental relationships between eating behavior and symptoms of anxiety and depression. Eat Weight Disord. 2019 Oct;24(5):887-895. doi: 10.1007/s40519-017-0445-2.
46. Kjeldgaard HK, Eberhard-Gran M, Benth JŠ, Vikanes ÅV. Hyperemesis gravidarum and the risk of emotional distress during and after pregnancy. Arch Womens Ment Health. 2017 Dec;20(6):747-756. doi: 10.1007/s00737-017-0770-5.
47. Sims M, Lipford KJ, Patel N, Ford CD, Min YI, Wyatt SB. Psychosocial Factors and Behaviors in African Americans: The Jackson Heart Study. Am J Prev Med. 2017 Jan;52(1S1):S48-S55. doi: 10.1016/j.amepre.2016.09.020.
48. Mackay DF, Anderson JJ, Pell JP, Zammit S, Smith DJ. Exposure to tobacco smoke in utero or during early childhood and risk of hypomania: Prospective birth cohort study. Eur Psychiatry. 2017 Jan;39:33-39. doi: 10.1016/j.eurpsy.2016.06.001.
49. Woolhouse H, James J, Gartland D, McDonald E, Brown SJ. Maternal depressive symptoms at three months postpartum and breastfeeding rates at six months postpartum: Implications for primary care in a prospective cohort study of primiparous women in Australia. Women Birth. 2016 Aug;29(4):381-7. doi: 10.1016/j.wombi.2016.05.008.
50. Moran P, Romaniuk H, Coffey C, Chanen A, Degenhardt L, Borschmann R, Patton GC. The influence of personality disorder on the future mental health and social adjustment of young adults: a population-based, longitudinal cohort study. Lancet Psychiatry. 2016 Jul;3(7):636-45. doi: 10.1016/S2215-0366(16)30029-3.
51. Yi SW. Depressive Symptoms on the Geriatric Depression Scale and Suicide Deaths in Older Middle-aged Men: A Prospective Cohort Study. J Prev Med Public Health. 2016 May;49(3):176-82. doi: 10.3961/jpmph.16.012.
52. Rossi R, Zammit S, Button KS, Munafò MR, Lewis G, David AS. Psychotic Experiences and Working Memory: A Population-Based Study Using Signal-Detection Analysis. PLoS One. 2016 Apr 27;11(4):e0153148. doi: 10.1371/journal.pone.0153148.
53. Bruins J, Pijnenborg MG, Bartels-Velthuis AA, Visser E, van den Heuvel ER, Bruggeman R, Jörg F. Cannabis use in people with severe mental illness: The association with physical and mental health--a cohort study. A Pharmacotherapy Monitoring and Outcome Survey study. J Psychopharmacol. 2016 Apr;30(4):354-62. doi: 10.1177/0269881116631652.
54. Gage SH, Hickman M, Heron J, Munafò MR, Lewis G, Macleod J, Zammit S. Associations of cannabis and cigarette use with psychotic experiences at age 18: findings from the Avon Longitudinal Study of Parents and Children. Psychol Med. 2014 Dec;44(16):3435-44. doi: 10.1017/S0033291714000531.
55. Hung YN, Yang SY, Huang MC, Lung FW, Lin SK, Chen KY, Kuo CJ, Chen YY. Cancer incidence in people with affective disorder: nationwide cohort study in Taiwan, 1997-2010. Br J Psychiatry. 2014 Sep;205(3):183-8. doi: 10.1192/bjp.bp.114.144741.
56. Dorrington S, Zammit S, Asher L, Evans J, Heron J, Lewis G. Perinatal maternal life events and psychotic experiences in children at twelve years in a birth cohort study. Schizophr Res. 2014 Jan;152(1):158-63. doi: 10.1016/j.schres.2013.11.006.
57. Artaud F, Dugravot A, Sabia S, Singh-Manoux A, Tzourio C, Elbaz A. Unhealthy behaviours and disability in older adults: three-City Dijon cohort study. BMJ. 2013 Jul 23;347:f4240. doi: 10.1136/bmj.f4240.
58. Sun WJ, Xu L, Chan WM, Lam TH, Schooling CM. Are depressive symptoms associated with cardiovascular mortality among older Chinese: a cohort study of 64,000 people in Hong Kong? Am J Geriatr Psychiatry. 2013 Nov;21(11):1107-15. doi: 10.1016/j.jagp.2013.01.048.
59. Jackowska M, Hamer M, Carvalho LA, Erusalimsky JD, Butcher L, Steptoe A. Short sleep duration is associated with shorter telomere length in healthy men: findings from the Whitehall II cohort study. PLoS One. 2012;7(10):e47292. doi: 10.1371/journal.pone.0047292.
60. Gale CR, Batty GD, Osborn DP, Tynelius P, Whitley E, Rasmussen F. Association of mental disorders in early adulthood and later psychiatric hospital admissions and mortality in a cohort study of more than 1 million men. Arch Gen Psychiatry. 2012 Aug;69(8):823-31. doi: 10.1001/archgenpsychiatry.2011.2000.

***No sufficient data***

1. Schneider B, Lukaschek K, Baumert J, Meisinger C, Erazo N, Ladwig KH. Living alone, obesity, and smoking increase risk for suicide independently of depressive mood findings from the population-based MONICA/KORA Augsburg cohort study. J Affect Disord. 2014 Jan;152-154:416-21. doi: 10.1016/j.jad.2013.10.007.
2. Meng X, D'Arcy C. The projected effect of risk factor reduction on major depression incidence: a 16-year longitudinal Canadian cohort of the National Population Health Survey. J Affect Disord. 2014 Apr;158:56-61. doi: 10.1016/j.jad.2014.02.007.
3. McGrath JJ, Alati R, Clavarino A, Williams GM, Bor W, Najman JM, Connell M, Scott JG. Age at first tobacco use and risk of subsequent psychosis-related outcomes: A birth cohort study. Aust N Z J Psychiatry. 2016 Jun;50(6):577-83. doi: 10.1177/0004867415587341.
4. Vulser H, Wiernik E, Tartour E, Thomas F, Pannier B, Czernichow S, Hanon O, Simon T, Simon JM, Ducolombier C, Consoli SM, Danchin N, Limosin F, Lemogne C. Smoking and the Association Between Depressive Symptoms and Absolute Neutrophil Count in the Investigations Préventives et Cliniques Cohort Study. Psychosom Med. 2015 Nov-Dec;77(9):1039-49. doi: 10.1097/PSY.0000000000000243.
5. Alibekova R, Huang JP, Lee TS, Au HK, Chen YH. Effects of smoking on perinatal depression and anxiety in mothers and fathers: A prospective cohort study. J Affect Disord. 2016 Mar 15;193:18-26. doi: 10.1016/j.jad.2015.12.027. Epub 2015 Dec 25. Erratum in: J Affect Disord. 2020 Sep 1;274:1218. doi: 10.1016/j.jad.2020.05.135.
6. Cooper J, Borland R, Yong HH, Fotuhi O. The impact of quitting smoking on depressive symptoms: findings from the International Tobacco Control Four-Country Survey. Addiction. 2016 Aug;111(8):1448-56. doi: 10.1111/add.13367.
7. Raffetti E, Donato F, Forsell Y, Galanti MR. Longitudinal association between tobacco use and the onset of depressive symptoms among Swedish adolescents: the Kupol cohort study. Eur Child Adolesc Psychiatry. 2019 May;28(5):695-704. doi: 10.1007/s00787-018-1237-6.
8. McGihon RE, Burns RJ, Deschênes SS, Schmitz N. Longitudinal associations between number of cigarettes per day and depressive symptoms in adult smokers with type 2 diabetes: A path analysis approach. J Psychosom Res. 2019 Oct;125:109737. doi: 10.1016/j.jpsychores.2019.109737.
9. Hassanzadah M, Bitar AH, Khanfar NM, Khasawneh FT, Lutfy K, Shankar GS. A retrospective cohort study of the prevalence of anxiety and agitation in schizophrenic smokers and the unmet needs of smoking cessation programs. Medicine (Baltimore). 2019 Oct;98(40):e17375. doi: 10.1097/MD.0000000000017375.
10. Lorenz DR, Misra V, Gabuzda D. Transcriptomic analysis of monocytes from HIV-positive men on antiretroviral therapy reveals effects of tobacco smoking on interferon and stress response systems associated with depressive symptoms. Hum Genomics. 2019 Nov 28;13(1):59. doi: 10.1186/s40246-019-0247-x.
11. de Azevedo Cardoso T, Jansen K, Mondin TC, Pedrotti Moreira F, de Lima Bach S, da Silva RA, de Mattos Souza LD, Balanzá-Martínez V, Frey BN, Kapczinski F. Lifetime cocaine use is a potential predictor for conversion from major depressive disorder to bipolar disorder: A prospective study. Psychiatry Clin Neurosci. 2020 Aug;74(8):418-423. doi: 10.1111/pcn.13012.
12. Perry BI, Stochl J, Upthegrove R, Zammit S, Wareham N, Langenberg C, Winpenny E, Dunger D, Jones PB, Khandaker GM. Longitudinal Trends in Childhood Insulin Levels and Body Mass Index and Associations With Risks of Psychosis and Depression in Young Adults. JAMA Psychiatry. 2021 Apr 1;78(4):416-425. doi: 10.1001/jamapsychiatry.2020.4180.
13. da Silva AHS, de Freitas LA, Shuhama R, Del-Ben CM, Vedana KGG, Martin IDS, Zanetti ACG. Family environment and depressive episode are associated with relapse after first-episode psychosis. J Psychiatr Ment Health Nurs. 2021 Dec;28(6):1065-1078. doi: 10.1111/jpm.12735.
14. Rabenstein A, Catarino CB, Rampeltshammer V, Schindler D, Gallenmüller C, Priglinger C, Pogarell O, Rüther T, Klopstock T. Smoking and alcohol, health-related quality of life and psychiatric comorbidities in Leber's Hereditary Optic Neuropathy mutation carriers: a prospective cohort study. Orphanet J Rare Dis. 2021 Mar 11;16(1):127. doi: 10.1186/s13023-021-01724-5.
15. Chichetto NE, Kundu S, Freiberg MS, Koethe JR, Butt AA, Crystal S, So-Armah KA, Cook RL, Braithwaite RS, Justice AC, Fiellin DA, Khan M, Bryant KJ, Gaither JR, Barve SS, Crothers K, Bedimo RJ, Warner A, Tindle HA; Veterans Aging Cohort Study. Association of Syndemic Unhealthy Alcohol Use, Smoking, and Depressive Symptoms on Incident Cardiovascular Disease among Veterans With and Without HIV-Infection. AIDS Behav. 2021 Sep;25(9):2852-2862. doi: 10.1007/s10461-021-03327-4.
16. Chaplin AB, Smith N, Jones PB, Khandaker GM. Direction of association between Cardiovascular risk and depressive symptoms during the first 18 years of life: A prospective birth cohort study. J Affect Disord. 2021 Sep 1;292:508-516. doi: 10.1016/j.jad.2021.05.094.
